# One‐Pot Chemical Protein Synthesis Utilizing Fmoc‐Masked Selenazolidine to Address the Redox Functionality of Human Selenoprotein F**

**DOI:** 10.1002/chem.202200279

**Published:** 2022-02-19

**Authors:** Zhenguang Zhao, Reem Mousa, Norman Metanis

**Affiliations:** ^1^ Institute of Chemistry The Hebrew University of Jerusalem Jerusalem 9190401 Israel; ^2^ The Center for Nanoscience and Nanotechnology The Hebrew University of Jerusalem Jerusalem 9190401 Israel; ^3^ Casali Center for Applied Chemistry The Hebrew University of Jerusalem Jerusalem 9190401 Israel

**Keywords:** chemical protein synthesis, protein folding, redox potential, selenocysteine, selenoprotein

## Abstract

Human SELENOF is an endoplasmic reticulum (ER) selenoprotein that contains the redox active motif CXU (C is cysteine and U is selenocysteine), resembling the redox motif of thiol‐disulfide oxidoreductases (CXXC). Like other selenoproteins, the challenge in accessing SELENOF has somewhat limited its full biological characterization thus far. Here we present the one‐pot chemical synthesis of the thioredoxin‐like domain of SELENOF, highlighted by the use of Fmoc‐protected selenazolidine, native chemical ligations and deselenization reactions. The redox potential of the CXU motif, together with insulin turbidimetric assay suggested that SELENOF may catalyze the reduction of disulfides in misfolded proteins. Furthermore, we demonstrate that SELENOF is not a protein disulfide isomerase (PDI)‐like enzyme, as it did not enhance the folding of the two protein models; bovine pancreatic trypsin inhibitor and hirudin. These studies suggest that SELENOF may be responsible for reducing the non‐native disulfide bonds of misfolded glycoproteins as part of the quality control system in the ER.

## Graphical abstract

**The one‐pot chemical synthesis** of the thioredoxin‐like domain of the ER human selenoprotein F using novel Fmoc‐protected selenazolidine is reported. This allowed us to perform different *in vitro* biological characterizations, including redox potential determination, insulin turbidimetric assay, and the effect on the oxidative folding of Cys‐rich protein models. Our results support that SELENOF acts as a reductase for non‐native disulfide bonds in the ER.

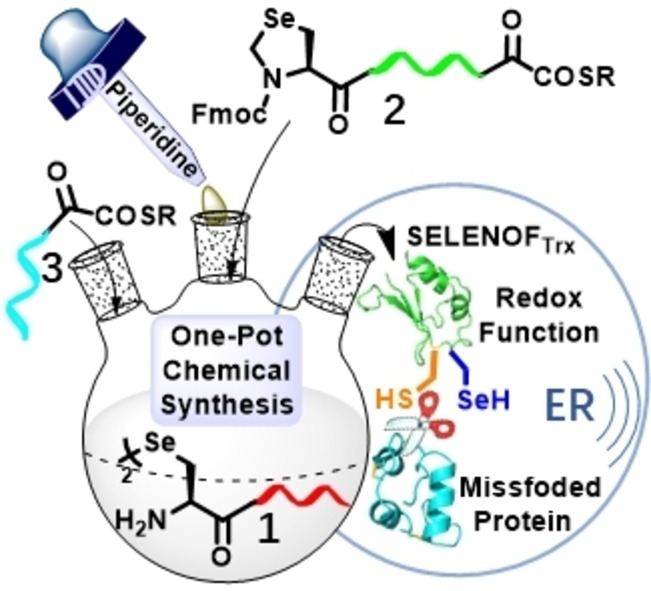

Selenium is an essential trace element for mammals and human health.^[1]^ The major role of selenium was attributed to its presence in a variety of functional selenoproteins, proteins containing the 21^st^ proteinogenic amino acid, selenocysteine (Sec, U).^[2]^ While previously the majority of research focused on aspects of nutritional Se deficiency or toxicity, recently more and more studies are directed to explore the functionality of selenoproteins, and their impact to human health and disease.^[3]^ The challenge in the characterization of these proteins underlay behind the diverse limitations in their biological and chemical preparation.^[2c]^ Among these poorly studied proteins is selenoproteins F, SELENOF. Human SELENOF (known also as Sep15) is a 15‐kDa endoplasmic reticulum (ER) selenoprotein (seven of the 25 known human selenoproteins are ER‐localized).^[4]^ SELENOF is highly expressed in various tissues, such as prostate, liver, kidney, and testes.^[5]^ NMR structural analysis of the fruit‐fly Sep15 (which is not a selenoprotein; the Sec is naturally replaced by Cys), together with a Sec‐to‐Cys mutant of another ER selenoprotein, mouse SELM (a homolog of human selenoprotein M, SELENOM), suggested that they are homologues to one another, and form a distinct selenoprotein family in the thioredoxin (Trx) superfamily.[^5^, ^6^] The two proteins, SELM and fruit‐fly Sep15 shared two major domains, the first is the signal sequence that is responsible for directing the protein into the ER, which is subsequently cleaved during protein maturation.^[7]^ The second is a common Trx‐like domain (with the characteristic α/β‐fold) that contains the redox‐active motif (typically a CXXC motif, C = Cys, X = any amino acid), suggesting they are involved in the thiol‐disulfide‐like interchange reactions and undergo reversible formation of a selenylsulfide (Se−S) bonds.[^5^, ^6^] Furthermore, human SELENOF carries unusual redox motif of CXU compared to the highly conserved motif CXXU in other selenoproteins, such as in SELENOM.^[6]^ Additionally, SELENOF possesses a distinct Cys‐rich domain in the N‐terminal, which is responsible for the tight binding of SELENOF with its partner protein UDP‐glucose:glycoprotein glyucosyltransferase (UGGT).^[8]^ UGGT is an essential regulator for quality control of *N*‐linked glycoprotein folding in the ER. This 170‐kDa enzyme catalyzes the transfer of the glucose moiety from UDP‐glucose to the terminal high‐mannose type oligosaccharide of partially misfolded glycoproteins, ensuring their retention in the ER for a second cycle by the calnexin (CNX) quality control pathway.^[9]^ The role of SELENOF in this cycle is not fully understood, however, the tight binding between SELENOF and UGGT (in a ratio of 1 : 1) with a *K*_d_ of 20 nM, implies that it may be either participating in modulating the UGGT enzymatic activity or involved in the formation/reduction of disulfide bonds of the UGGT substrates.^[9]^ More recent study has suggested that SELENOF acts as a gatekeeper that blocks the secretion of misfolded disulfide‐rich glycoproteins allowing them to participate in an additional maturation cycle in the ER.^[10]^ Additionally, SELENOF was suggested to be engaged in the unfolded protein response (UPR) signaling pathway, where it was highly expressed by a response treatment for unfolded proteins accumulation in the ER.^[11]^

In order to shed more light on the exact function of SELENOF, especially its role in protein folding, and to avoid any interference of Cys‐rich domain, which is known to bind the UGGT,^[8]^ we decided to focus on its Trx‐like domain with the correct active site (CXU motif).

Recent advances in protein expression and new approaches developed for accessing selenoproteins^[12]^ hold good promise, still chemical protein synthesis (CPS) is a powerful alternative that offers various tools to access selenoproteins, or any proteins with rare or unnatural amino acids.[^2c^, ^13^] Native chemical ligation (NCL) has become the most widely used approach for chemoselective linking between unprotected peptide segments in aqueous solutions for the preparation of large proteins. The desulfurization of Cys and deselenization of Sec were developed to expands NCL to other sites not restricted to Cys/Sec.^[14]^ While desulfurization requires protecting the thiols of natural Cys residues in the sequence, deselenization is chemoselective and can be performed in the presence of unprotected Cys residues.^[14c]^ Further, the deselenization of Sec under anaerobic conditions yield Ala, while it provides Ser under oxidative conditions.[^14c^, ^15^] Further, in case of a multistep NCL reactions, thiazolidine (Thz)^[16]^ and selenazolidine (Sez, Z)^[17]^ (protected forms of N‐terminal Cys and Sec, respectively) were utilized to avoid undesired intramolecular cyclization in middle segment peptides bearing a C‐terminal thioester.[^17^, ^18^] Although Sez has been utilized for one‐pot CPS,[^17^, ^18^, ^19^] in some cases we observe that Sez is not stable during the deselenization step, which may limit its use in CPS.^[19]^ While *p*‐methoxybenzyl (Mob) can be an alternative protecting group of Sec, previous studies in our research group indicated that this protecting group is not stable during the deselenization or desulfurization reactions.^[19a]^ Furthermore, the harsh conditions of deprotection step by TFA and 2,2’‐dithiobis(5‐nitropyridine) (DTNP)^[20]^ are not compatible with our one‐pot synthetic approach. Previously, different protecting groups were developed to increase the stability of Thz during hydrazide oxidation to azides,^[21]^ among these were *tert*‐butyldisulfanylethyloxycarbonyl (Tbeoc),^[21]^ 9‐fluorenylmethoxy‐carbonyl (Fmoc)^[22]^ and trifluoroacetyl (Tfa) groups.^[23]^ It is worth noting that Fmoc‐Cys was recently used to mask the N‐terminal Cys for one‐pot CPS.^[24]^ Following ligation, Fmoc is removed by 20 % piperidine in aqueous solution.^[24]^ Hence, we decided to test Fmoc as a protecting group of Sez during CPS, which prevented the undesired deselenization at position 96 in the redox motif, and provided milligram quantities of the Trx‐like domain of SELENOF. This allowed us to characterize the Trx‐like domain of SELENOF, including redox potential determination and its capability to catalyze disulfide bonds reduction or enhance oxidative protein folding.

The Trx‐like domain sequence of SELENOF, SELENOF(89‐165) (in short SELENOF_Trx_), contains the Cys94 and Sec96 found in the CGU motif. Therefore, we decided to synthesize SELENOF_Trx_ from three segments and two NCL reactions (Figure 2a and b),^[25]^ with Gly95‐Sec96 and Ile140‐Ala141 as the ligation junctions. To allow for sequential ligations, Ala141 was temporary replaced with Sec,[^15b^, ^26^] while the native Sec96 was replaced with the protected derivative Sez to prevent intermolecular cyclization (Scheme S2, Figure S2).^[17]^ The corresponding C‐terminal peptides SELENOF(141‐165)(A141U) was synthesized by Fmoc‐SPPS, purified and characterized by HPLC and ESI‐MS (Scheme S1, Figure S1). Both SELENOF(96‐140)(Sec96Sez)‐COSR and SELENOF(89‐95)‐COSR (Schemes S2 and S3), bearing a C‐terminal thioester^[27]^ were prepared similarly by standard stepwise Fmoc‐SPPS (further details for the syntheses can be found in Supporting Information 4.3 and Figures S2–S4).[^27a^, ^28^] The ligation between SELENOF(96‐140)(Sec96Sez)‐COSR and SELENOF(141‐165)(A141U) was performed at 37 °C for 18 h in the presence of TCEP and sodium ascorbate^[29]^ to yield SELENOF(96‐165)(Sec96Sez/A141U) (Figure S5). To convert Sec141 to Ala, the purified ligated product was subjected to deselenization reaction with TCEP at pH∼5, and under anaerobic conditions.[^15b^, ^26^] Unfortunately, we observed that Sez96 was not stable under these conditions, and significant ring opening and deselenization occurred after 30 min to yield the undesired Sec96Ala side‐product. Despite our efforts to optimize the selectivity of this reaction, the doubly deselenized side‐product, with undesired Ala96 in addition to the desired Ala141, predominate (with as high as ∼60 % in many cases) (Figure S5). Aiming to enhance the stability of Sez under the deselenization reaction conditions, we decided to check if an N‐terminal protected form of Sez, specifically with Tfa or Fmoc, would provide higher yields of the desired product. The Tfa/Fmoc protecting groups can be removed post deselenization step (Scheme S6, Figure S9).

We first tested Tfa as a protecting group of Sez with model peptide bearing N‐terminal Tfa‐Sez residue (Tfa‐Sez‐LYRAG‐NH_2_). However, we observed a serious epimerization of Sec during the Tfa‐deprotection step (normally performed at pH 10–11, see the detail in the Supporting Information 4.4.2.1, Figure S7). Next, Fmoc‐Sez was tested in the model peptide Fmoc‐Sez‐LYRAG‐NH_2_. When the model peptide was treated with 20 % piperidine (Pip) in phosphate buffer at pH 10, we obtained the peptide ULYRAG‐NH_2_ within 5 h, indicating that a one‐pot Fmoc deprotection and Sez opening occurred. The deselenization of the product, ULYRAG‐NH_2_, with 100 equiv. of TCEP, provided only *l*‐enantiomer product (*l*)‐ALYRAG‐NH_2_ alongside TCEP=Se adduct (Figure 1). Co‐injection of the deselenization reaction mixture with authentic (*l*)‐ALYRAG‐NH_2_ and (*d*)‐ALYRAG‐NH_2_, which were separately synthesized, suggested that no epimerization of Sec residue occurred during the Fmoc deprotection step (Figure 1). Hence, we decided to use Fmoc‐Sez for the preparation of the Trx‐like domain of SELENOF. The synthesis of all peptide segments needed for the preparation of Trx‐like domain of SELENOF is provided in the Supporting Information. Ligation between SELENOF(96‐140)(Sec96FmocSez)‐COSR and SELENOF(141–165)(A141U) was performed in the presence of TCEP (4 equiv., which were added in portions, see Supporting Information) at 37 °C. After 10 h the reaction was completed (Figures 2b, c and S6), where Fmoc‐Sez remain intact under the NCL conditions, as expected (Figures 2c and S6). Next, without a purification step, the pH was adjusted to 5.5 and the deselenization of SELENOF(96–165)(U96FmocSez/A141U) was performed under anaerobic conditions using 50 equiv TCEP at 37 °C. After 13 h (Figures 2c and S8), the reaction mixture was treated with 20 % Pip in phosphate buffer containing 0.1 M sodium ascorbate at pH 10.[^22^, ^24^] Without NH_2_OMe,^[17]^ Cu ions^[18]^ or any additive,^[30]^ we were delighted to find that these conditions provided the Fmoc deprotection and Sez opening in one step within 5 h (Figures 2b, c and S9).

**Figure 1:**
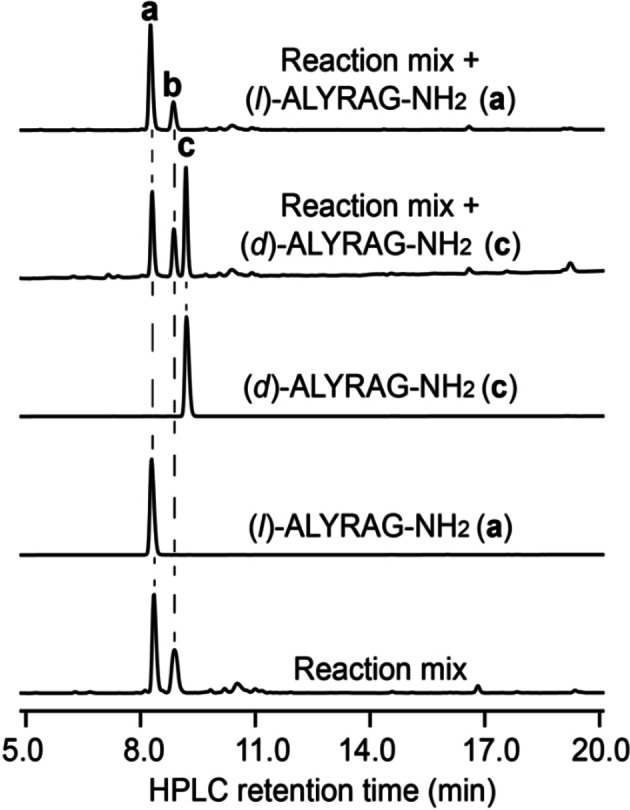
Epimerization study for Fmoc‐protected Sez containing model peptide. Deprotection was performed in phosphate buffer at pH 10 in the presence of 20 % Pip, followed by a deselenization at pH 5 using 100 equiv. of TCEP. **a** is (*l*)‐ALYRAG‐NH_2_, **b** is TCEP=Se adduct, and **c** is (*d*)‐ALYRAG‐NH_2_.

**Figure 2:**
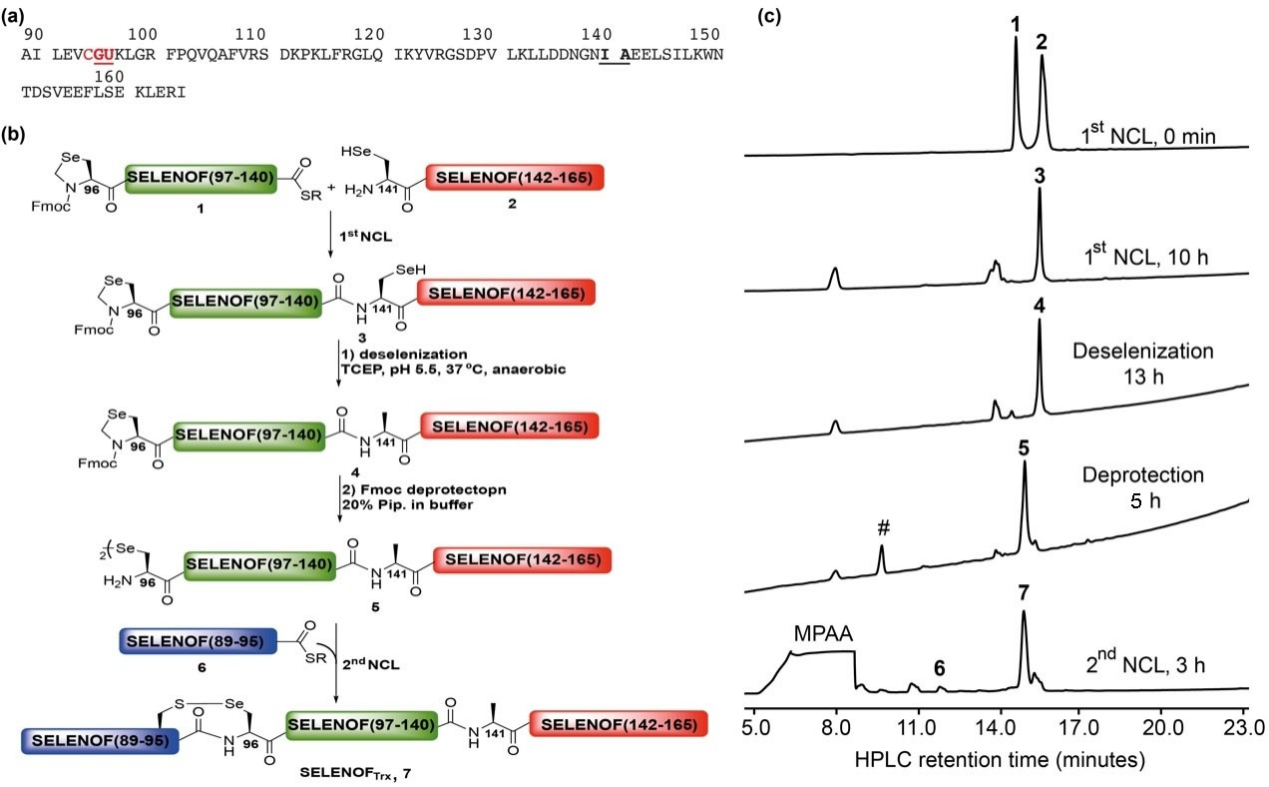
a) The sequence of the Trx‐like domain of SELENOF, SELENOF_Trx_, the CGU motif is highlighted, and the ligation junctions are underline; b) synthetic approach for SELENOF_Trx_; c) HPLC chromatograms for the one‐pot preparation of SELENOF_Trx_. #=Fmoc‐Pip adduct.

The presence of 20 % Pip in the reaction was compatible with the following NCL reaction conditions, as Pip is fully protonated at neutral pH, preventing any side reaction with the thioester.^[24]^ Without further purification step, SELENOF(89‐95)‐COSR was dissolved in phosphate buffer at pH 7 containing 0.05 M TCEP, 0.1 M sodium ascorbate and 0.1 mM MPAA and was added directly for the second NCL reaction. After two NCL reactions, a deselenization step, deprotection of Fmoc and Sez opening, all in one‐pot, the final product, SELENOF_Trx_, was isolated in 23 % overall yield (Figures 2b, c, and S10, S13 for HR‐MS).

Following synthesis and purification, SELENOF_Trx_ was allowed to fold in phosphate buffer at pH 9, where basic conditions were necessary for dissolving this protein (see Supporting Information 4.5). Next, structural analysis using CD showed that the protein is folded and contains secondary structure features of the α/β‐fold (Figure S11). Compared to human SELENOM, which was previously synthesized and structurally analyzed in our lab,^[19]^ we can clearly see that both proteins share similar characteristics with each other and with Trx proteins superfamily.

Since SELENOF is a member of the thiol‐disulfide oxidoreductase family, it is important to study its role in the formation, reduction, or isomerization of disulfide bonds. Hence the determination of SELENOF redox potential and the comparison to other well‐characterized oxidoreductases will assist in understanding its chemical ability to accept or donate electrons, and more specifically, its role in the protein folding in the ER. To determine SELENOF redox potential, we used protein‐protein redox equilibria,^[31]^ in which equimolar concentration of oxidized and folded form of SELENOF_Trx_ was incubated with reduced *E.coli* Trx under anaerobic conditions to allow the reduction‐oxidation reaction to reach equilibrium (see Supporting Information 4.6 for details).^[31]^ The proteins reached the equilibrium within 8 min (Figure 3a) as judged by HPLC (Figure 3b), and the redox potential of the SELENOF_Trx_ was determined using Nernst equation to give −256.3±0.8 mV (Figure 3c). This redox potential is lower than the reported redox potential of the fruit‐fly Sep15 homolog (with a CTC motif, determined as −225 mV).^[32]^ When the redox potential of SELENOF_Trx_ was compared to other members from oxidoreductase family it showed much lower value than the protein‐disulfide isomerase (PDI) (−175 mV)^[31]^ and closer to the *E. coli* Trx (−270 mV, which was taken as the reference)^[33]^ (Figure 3c). Our result is consistent with the reported redox potential of the Sec‐substituted *E. coli* Grx3 analogs (Grx3(C11 U) and Grx3(C14U), with UXXC and CXXU motifs, and redox potential of −260 and −275 mV, respectively).^[34]^ This may suggest that SELENOF is potentially involved in the disulfide bonds reduction, and does not behave as oxidase or isomerase in the thiol‐disulfide exchange reactions (Figure 3c). These results encouraged us to further investigate the role of SELENOF in disulfide bonds reduction and protein folding.

**Figure 3:**
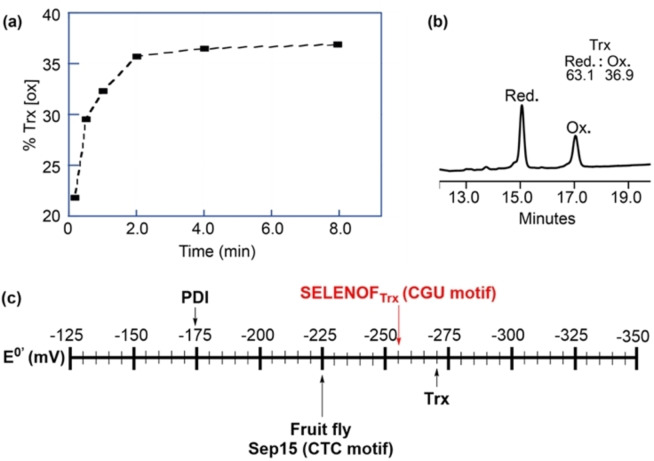
a) Formation of oxidized Trx as a function of time in the redox equilibria of reduced *E. coli* Trx and oxidized SELENOF_Trx_. b) HPLC chromatogram of the reduced and oxidized species of *E. coli* Trx after reaching equilibrium; c) the standard state redox potentials of some of the thiol‐disulfide oxidoreductases. Indicated here the redox potential of SELENOF_Trx_ (*red*), the *E. coli* protein disulfide isomerase (PDI), *E. coli* Trx, and fruit‐fly Sep15 (with CTC motif).[^31^, ^32^]

Next, we tested if SELENOF_Trx_ may act as a reductant (with a Trx‐like activity), so the insulin turbidimetric assay was examined.^[35]^ In this assay, which was developed by Holmgren, the ability of a protein to catalyze the reduction of disulfide bonds in insulin by DTT is studied. Fresh mixture of insulin and SELENOF_Trx_ was prepared in phosphate buffer at pH 7, and the reaction was initiated by the addition of DTT to the cuvette and scanned at 650 nm for 120 min (see the detail in the Supporting Information 4.7). The results demonstrate that SELENOF_Trx_ catalyzes the cleavage of the two interchain disulfide bridges of insulin by DTT, where white precipitation was formed from the insoluble free B chain of insulin. This indicated that SELENOF may act as a reductant *in vivo* that catalyzes disulfide reduction. Still, *E. coli* Trx showed higher activity than SELENOF perhaps due to its lower redox potential (Figure 4).

**Figure 4:**
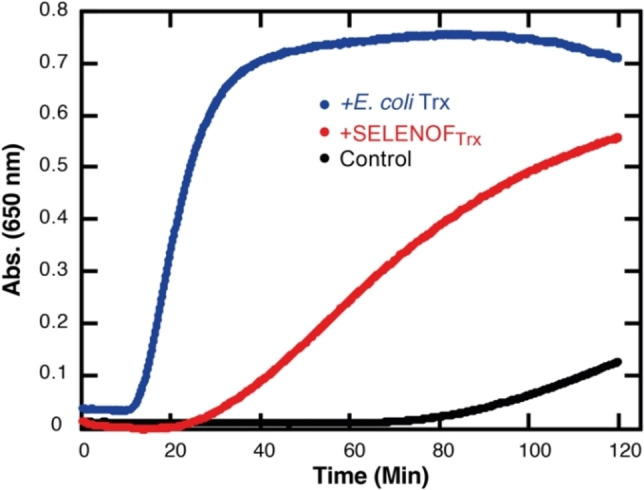
Turbidimetric assay of insulin reduction by *E. coli* Trx (*blue*) and SELENOF_Trx_ (*red*) in the presence of DTT. In a cuvette, insulin (0.13 M) and SELENOF_Trx_ or *E. coli* Trx (at 7.8 μM each) were incubated first in phosphate buffer at pH 7 and room temperature, followed by the addition of DTT (0.33 mM) to initiate the reaction. The absorbance at 650 nm was followed by UV spectrophotometer. In the control experiment only insulin and DTT were present (*black*).

To further investigate the role of SELENOF in protein folding we chose to explore the effect of SELENOF_Trx_ in the oxidative folding of the two well‐studied proteins; bovine pancreatic trypsin inhibitor (BPTI) and hirudin, which represent opposite folding models to many disulfide‐rich proteins.^[36]^ Weissman and Kim studied BPTI folding in the presence of PDI,^[37]^ which increased dramatically both the yield and the rate of the native state formation. In BPTI folding, PDI functions as a catalyst for the rate‐determining intramolecular rearrangement from the trapped intermediates N’ and N* to form $N_{SH}^{SH}$ , the direct precursor to N. Furthermore, previously the Sec‐substituted BPTI^[38]^ and hirudin^[39]^ analogs, as well as other proteins^[40]^ have been investigated, and in all cases, Sec was found to enhance the folding kinetics and yields of these proteins. We have initiated our studies on BPTI by following previously reported folding conditions.^[40]^ The folding of BPTI was tested in the presence and the absence of the SELENOF_Trx_ to allow a direct comparison, using catalytic amount of SELENOF_Trx_ (5 μM) and the redox pair GSSG/GSH under anaerobic conditions. We found that in the presence or the absence of SELENOF_Trx_, BPTI folded identically through its characteristic folding features,[^37^, ^41^] indicating that SELENOF has no impact on its folding rate and yield (Figure S12a and b). We also checked the folding of hirudin as an alternative candidate, where it folds to the native state through heterogenous pathway that involve the formation of non‐native intermediates.^[42]^ However, under anaerobic conditions and in the presence of catalytic amounts of SELENOF_Trx_, hirudin folded through the established pathway[^39^, ^42^] showing no difference when compared to the control experiment (Figure S12c and d).

These results may indicate that SELENOF is only specific to misfolded glycoproteins, as it forms a tight complex with UGGT,^[8]^ the folding sensor in the quality control system of glycoproteins folding. All together, the low redox potential, and insulin turbidimetric assay, further support that SELENOF is not a PDI‐like enzyme, but rather it acts as a Trx‐like protein catalyzing disulfide bonds reduction.

In summary, our main goal in this study was to synthesize and characterize the poorly studied ER selenoprotein F, aiming to understand more about its function in general and its role in protein folding in particular. We chose to focus on the Trx‐like domain of SELENOF (SELENOF_Trx_), which contains the unusual active site motif CGU, and was proposed to take part in the folding of misfolded proteins in the ER.

Here, we develop Fmoc‐protected selenazolidine (Fmoc‐Sez) as a protected form of Sec for middle peptide segments bearing C‐terminal thioester, which significantly enhanced the stability of Sez during deselenization reaction. The removal of Fmoc and Sez opening could be achieved in one step by 20 % Pip in buffer, which is also compatible with following NCL reactions. The use of Fmoc‐protected Sez into the multi‐step chemical protein synthesis, enabled us to access SELENOF_Trx_ in two NCL reactions, a deselenization, Fmoc‐deprotection and Sez opening, in one‐pot approach. This strategy enhanced the efficiency and overall yield, allowing us to perform a battery of *in vitro* biological characterizations on SELENOF.

The redox potential of SELENOF_Trx_ and its ability to catalyze the reduction of disulfide bonds in insulin, suggest that SELENOF may function as a disulfide reductase for misfolded proteins. Furthermore, when SELENOF_Trx_ was included in the oxidative folding of the two model proteins, BPTI and hirudin, we did not observe any effect on their folding, further supporting its function as a Trx‐like protein and not as a PDI‐like or an oxidase‐like protein. It is plausible that SELENOF might target a restricted group of UGGT substrates,^[11]^ since UGGT is known to recognize partially folded/misfolded glycoproteins. This study brings us closer to dissecting the function of human SELENOF in the ER.

## Conflict of interest

The authors declare no conflict of interest.

## Supporting information

As a service to our authors and readers, this journal provides supporting information supplied by the authors. Such materials are peer reviewed and may be re‐organized for online delivery, but are not copy‐edited or typeset. Technical support issues arising from supporting information (other than missing files) should be addressed to the authors.

Supporting Information

## Data Availability

The data that support the findings of this study are available in the supplementary material of this article.
