# Supplementary material for: One‐Pot Chemical Protein Synthesis Utilizing Fmoc‐Masked Selenazolidine to Address the Redox Functionality of Human Selenoprotein F
Source: Chemistry. 2022 Feb 19;28(16):e202200279. doi: 10.1002/chem.202200279 (PMC9304195; doi:10.1002/chem.202200279)
Supplement: Supplementary file 1 — Supporting Information [file CHEM-28-0-s001.pdf]

Chemistry–A European Journal

Supporting Information

One-Pot Chemical Protein Synthesis Utilizing Fmoc-Masked Selenazolidine to Address the Redox Functionality of Human Selenoprotein F

Zhenguang Zhao, Reem Mousa, and Norman Metanis*

Table of content

1. Materials and Methods 3

2. High Performance Liquid Chromatography (HPLC) 3

3. Mass spectrometry (MS) and HR-MS 4

4. Experimental section 4

4.1. Fmoc-*L*-Sez-OH synthesis 4

4.2. Tfa-*L*-Sez-OH synthesis 5

4.3. Peptide synthesis 6

4.3.1. Synthesis of the model peptides Tfa/Fmoc-Sez-LYRAG-NH2 6

4.3.2. Synthesis of SELENOF(140-165)(A141U) 7

4.3.3. Synthesis of SELENOF(96-140)(Sec96FmocSez)-COSR and SELENOF(96-140)(Sec 96Sez)-COSR 9

4.3.4. Synthesis of SELENOF(89-95)-COSR 12

4.4. Native chemical ligation (NCL), Deselenization and deprotection reactions 14

4.4.1. 1st ligation reaction 14

4.4.2. Deselenization reactions 16

4.4.2.1. Testing the epimerization during the removal of Tfa group from Sez 16

4.4.2.2. Deselenization of SELENOF(96-165)(Sec96FmocSez/A141U) 18

4.4.3. Fmoc deprotection and Sez opening 19

4.4.4. 2nd ligation reaction 20

4.5. Circular Dichroism (CD) 21

4.6. Redox potential determination 22

4.7. Turbidimetric assay 23

4.8. Oxidative folding studies 23

4.9. HR-MS 25

5. NMR Results 26

6. References 31

1. Materials and Methods

Buffers for both ligation reactions and kinetic experiments were prepared using MilliQ water (Millipore, Merck). Ultrapure guanidinium chloride (Gn·HCl, Apollo Scientific Ltd, England) was used in all ligation buffers. $Na_2HPO_4 \cdot 12H_2O$, tris(2-carboxyethyl)phosphine hydrochloride (TCEP·HCl), 4-mercaptophenylacetic acid (MPAA), 2,2`-Dithiobis (5-nitropyridine) (DTNP), sodium ascorbate, *D,L*-dithiothreitol (DTT), triisopropylsilane (TIPS), acetylacetone (acac), oxidized and reduced *L*-Glutathione (GSSG/GSH) and *E.coli* Trx were purchased from Merck (Jerusalem, Israel). All Fmoc-amino acids were obtained from CS Bio Co. (Menlo Park, CA) or Matrix innovation (Quebec City, Canada), with the following side chain protecting groups: Arg(Pbf), Asp(OtBu), Glu(OtBu), Ser(tBu), Thr(tBu), Cys(Trt), Lys(Boc), Tyr(tBu), Asn(Trt). (Pbf = 2,2,4,6,7- pentamethyl-2,3-dihydrobenzofuran-5-sulfonyl). TentaGel® R RAM resin (loading 0.19 mmol/g) and 2-chlorotrityl chlorideresin (loading 0.4 mmol/g) were purchased from Rapp Polymer GmbH (Germany), GL Biochemical (China) or Chem-Impex (USA). *N,N,N',N'*-Tetramethyl-O-(6-chloro-1H-benzotriazol-1-yl)uronium hexafluorophosphate (HCTU) and Ethyl cyano(hydroxyimino)acetate (OxymaPure) were purchased from Luxembourg Biotechnologies Ltd. (Rehovot, Israel). All solvents: *N,N*-dimethylformamide (DMF), dichloromethane (DCM), acetonitrile (MeCN), *N,N*-diisopropylethyl amine (DIEA), piperidine (Pip), diethyl ether (Et_2O) and trifluoroacetic acid (TFA) were purchased from Bio-Lab (Jerusalem, Israel) and were peptide synthesis, HPLC or ULC-grade. Fmoc-Sec(MoB)-OH was synthesized as reported previously.[1]

2. High Performance Liquid Chromatography (HPLC)

The analytical analyses were performed on a reverse-phase Waters Alliance HPLC with UV detector (220 nm and 280 nm) using an X-Bridge C4 column (300 Å, 3.5 µm, 4.6 ×150 mm) and for oxidative folding experiment Atlantis T3 column (3 µm, 4.6 × 150 mm). Preparative and semi-preparative RP-HPLC was performed on a Waters LCQ150 system using XSelect C18 column (130 Å, 5 µm, 30 × 250 mm), X-Bridge BEH C4 (300 Å, 5 µm 19 × 150 mm) and X-Bridge BEH C4 (300 Å, 5 µm, 10 × 150 mm). Linear gradients of MeCN with 0.1% TFA (buffer B) and water with 0.1% TFA (buffer A) were used for all systems to elute bound peptides. The flow rates were

1 mL/min (analytical), 3.34 mL/min (semi-preparative), 10 mL/min and 20 mL/min (C4 preparative and C18 preparative, respectively).

3. Mass spectrometry (MS) and HR-MS

MS was performed on Thermo Scientific-LCQ Fleet Ion-Trap mass spectrometer. Peptides masses were calculated from the experimental mass to charge (m/z) ratios from the observed multiply charged species of a peptide using MagTran v1.03.

The HR-MS were recorded on a Q-ExactivePlus Orbitrap mass spectrometer (Thermo Scientific) with a ESI source and 140000 FWHM, in a method with AGC target set to 1E6, and scan range was 400-2800 m/z. The raw data was deconvoluted by MagTran v1.03 software.

4. Experimental section

4.1. Fmoc-*L*-Sez-OH synthesis

(*R*)-1,3-selenazolidine-4-carboxylic acid was prepared from *L*-selenocystine as was descried previously.[1] Next, cold solution of NaOH (1 equiv, 6 mmol) in water (31 mL) was added to the lyophilized crude mixture of (*R*)-1,3-selenazolidine-4-carboxylic acid at 0 ^{o}C. After 10 min, Fmoc-Osu (1.2 mmol, 1.2 equiv) in 1,4-dioxane (3 mL) was added dropwise at 0 ^{o}C over a period of 30 min. After 5 min stirring, the cooling bath was removed and the reaction stirred at room temperature for 13 h. The solution was acidified with 1N HCl and extracted with EtOAc. The organic layer was washed with 1N HCl, brine and dried over Na_2SO_4 and evaporated. The resulting crude was purified by flash column chromatography (60-120 mesh Silica gel, 1-4% MeOH in $CHCl_3$) to afford the desired product Fmoc-Selenazolidine (Fmoc-Sez-OH) (0.5 g, 41% yield for the two steps). ^{1}H NMR (400 MHz, DMSO-d_6) δ 13.14 (1H, brs, COO*H*), 7.90 (2H, phenyl, d, J_{HH} =7.5 Hz), 7.66 (2H, phenyl, m), 7.43 (2H, phenyl, t, J_{HH} =7.6 Hz), 7.33 (2H, phenyl, t, J_{HH} =7.0 Hz), 5.15 (1H, NC*H*CO), 4.76 (1H, OCH_2CH), 4.36 (2H, NCH_2Se and 2H, OCH_2CH), 3.29 (2H, CHCH_2Se) ppm (Figure S14). ^{13}C NMR (125.8 MHz, DMSO-d_6) δ 171.3, 153.6, 143.6 (rotamer), 140.7, 127.7, 127.2, 125.1, 120.2, 67.4-66.4 (rotamer), 63.0-62.1 (rotamer), 46.5, 38.1, 25.5-24.3 (rotamer) ppm (Figure S15).

4.2. Tfa-*L*-Sez-OH synthesis

(*R*)-1,3-selenazolidine-4-carboxylic acid was prepared from *L*-selenocystine as was descried previously.[1] The crude compound (1.3 g, 7.68 mmol) was dissolved in MeOH (30 mL) and cooled to – 10 °C, then $SOCl_2$ (1.1 mL, 14.57 mmol, 2 equiv) was added dropwise over a period of 10 min. The ice bath was removed, and the reaction mixture was refluxed for 17 h (85-90 °C). Then the reaction mixture was extracted twice with ethyl acetate, dried with Na_2SO_4 and evaporated by rotavap to yield (*R*)-1,3-selenazolidine-4-ester (0.8 mg, 61 %), which was used in the next step without further purification. Next, (*R*)-1,3-selenazolidine-4-ester (0.8 g, 4.38 mmol) was dissolved in DCM (5 mL), cooled to 0 °C and then Trifluoroacetic anhydride (1.24 mL, 8.76 mmol, 2 equiv) was added dropwise. After the anhydride addition was completed the cooling bath was removed and the reaction was stirred at room temperature for 15 h. The reaction mixture was quenched with Na_2CO_3 and extracted twice with DCM to yield Tfa-Sez-OMe (1 g, 76 %).

Tfa-Sez-OMe (0.67 mg, 2.31 mmol) was dissolved in dry DCM (10 mL) and the solution was cooled to -78 °C using liquid N_2 and acetone. Then, BBr_3 (6.9 mL, 3 equiv) was added dropwise to the solution for a period of 5 min, the ice bath removed, and the reaction was kept for 1.5 h at room temperature. When the reaction was over it was quenched carefully with 5 mL of water at 0 °C followed by adding acidic buffer (pH 2.5) to adjust the pH at 5. The reaction mixture was extracted three times with DCM, dried with Na_2SO_4 and evaporated. The crude product was purified with by flash column chromatography (0.04-0.06 mm Silica gel, EA/hexane (35/65) with 1% acetic acid) to afford the desired product Tfa-Selenazolidine (Tfa-Sez-OH) (0.44 g, 70%). The overall yield for the reaction is 40.5%. ^1H NMR (400 MHz, $CDCl_3$) δ 9.48 (1H, brs, COO*H*), 5.59-5.39 (1H, m, NC*H*CO), 5.13-4.97 (1H, m, NCH_2Se), 4.76-4.59 (1H, m, NCH_2Se), 3.58-3.44 (1H, m, CHCH_2Se), 3.40-3.34 (1H, m, CHCH_2Se) ppm (Figure S16). ^{13}C NMR (125.8 MHz, $CDCl_3$) δ 168.9, 156.1-155.0 (rotamer), 120.4-111.8 (rotamer), 63.9-62.9 (rotamer), 39.6-37.6 (rotamer), 25.8-23.4 (rotamer) ppm (Figure S17).

4.3. Peptide synthesis

General procedure for Fmoc-SPPS

Peptides were prepared manually or by using an automated peptide synthesizer (CS136XT, CS Bio Inc. CA) typically on 0.25 mmol scale. Fmoc-amino acids (2 mmol) were activated with HCTU (2 mmol) and DIEA (4 mmol) for 5 min and coupled for 25 min, with constant shaking. Fmoc deprotection step was carried out with 20% piperidine in DMF for 2 x 10 min, and DMF was used for washing the resin. Fmoc-Sec(Mob)-OH, Fmoc-Sez-OH, Boc-Sez-OH and Tfa-Sez-OH were coupled manually using DIC/OxymaPure activation method.[2]

4.3.1. Synthesis of the model peptides Tfa/Fmoc-Sez-LYRAG-NH_2

The model peptide was synthesized manually on TentaGel® R RAM resin (0.19 mmol/g, 0.25 mmol scale). The resin was swelled in DMF and DCM for 1 h and treated with 1 mmol of Fmoc-Gly-OH and 1 mmol of DIEA in DMF, which was coupled manually for 1.5 h. After draining, another fresh batch of Fmoc-Gly-OH and DIEA were added to the resin and shaken for additional 1.5 h. The unreacted resin was capped by 10 min shaking in 5 mL MeOH. The full synthesis we processed according to the general procedure for Fmoc-SPPS. Tfa/Fmoc-Sez-OH were coupled for 2 h (3.0 equiv Tfa/Fmoc-Sez-OH activated on ice for 5 min using 3.0 equiv OxymaPure and 2.9 equiv DIC) [2]. Following the SPPS the peptide was cleaved and deprotected using TFA: TIPS: water (94: 3: 3) cocktail. The peptide was purified using RP-HPLC (C18 column) to give pure of Tfa/Fmoc-Sez-LYRAG-NH_2. (Figure S18).

The sequence of the Trx-like domain of SELENOF, SELENOF$_{Trx}$; UniProt - 060613

```
 90        100        110        120        130        140        150
AI LEVCGUKLGR FPQVQAFVRS DKPKLFRGLQ IKYVRGSDPV LKLLDDNGNI AEELSILKWN
      160
TDSVEEFLSE KLERI
```

The Trx-like domain of SELENOF was synthesized using three segments, two ligation reaction and one deselenization reaction. The ligation sites are in bold and underlined. The active site redox motif CGU is shown in red. The detailed syntheses of the segments and ligation reaction are described below.

4.3.2. Synthesis of SELENOF(140-165)(A141U)

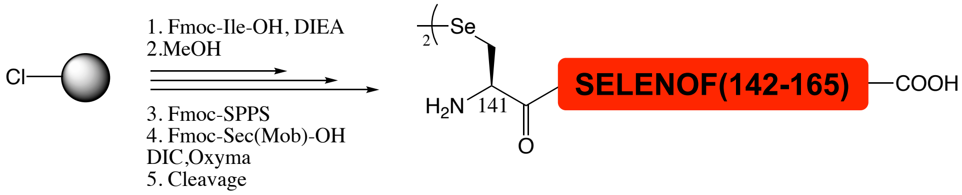

Scheme S1. Chemical synthesis of SELENOF(141-165)(A141U) by SPPS.

The synthesis of SELENOF(141-165)(A141U) was carried out on 2-chlorotrityl chloride resin (0.4 mmol/g, 0.25 mmol scale) on an automated peptide synthesizer. The resin was swelled in DMF and DCM for 1 h and treated with 1 mmol of Fmoc-Ile-OH and 1 mmol of DIEA in DMF, which was coupled manually for 1.5 h. After draining, another fresh batch of Fmoc-Ile-OH and DIEA were added to the resin and shaken for additional 1.5 h. The unreacted resin was capped by 10 min shaking in 5 mL MeOH. Ala141 was replaced with Sec, which was manually coupled for 2 h (3.0 equiv Fmoc-Sec(Mob)-OH activated on ice for 5 min using 3.0 equiv OxymaPure and 2.9 equiv DIC) [2] and treated with 20% of piperidine for Fmoc final deprotection. Following the SPPS the peptide was cleaved and deprotected using TFA: TIPS: water (94: 3: 3) cocktail and 2.0 equiv DTNP.[3] The peptide was purified using RP-HPLC (C18 column) to give pure fragment of SELENOF(141-165)(A141U) in ~39 % yield (Scheme S1, Figure S1).

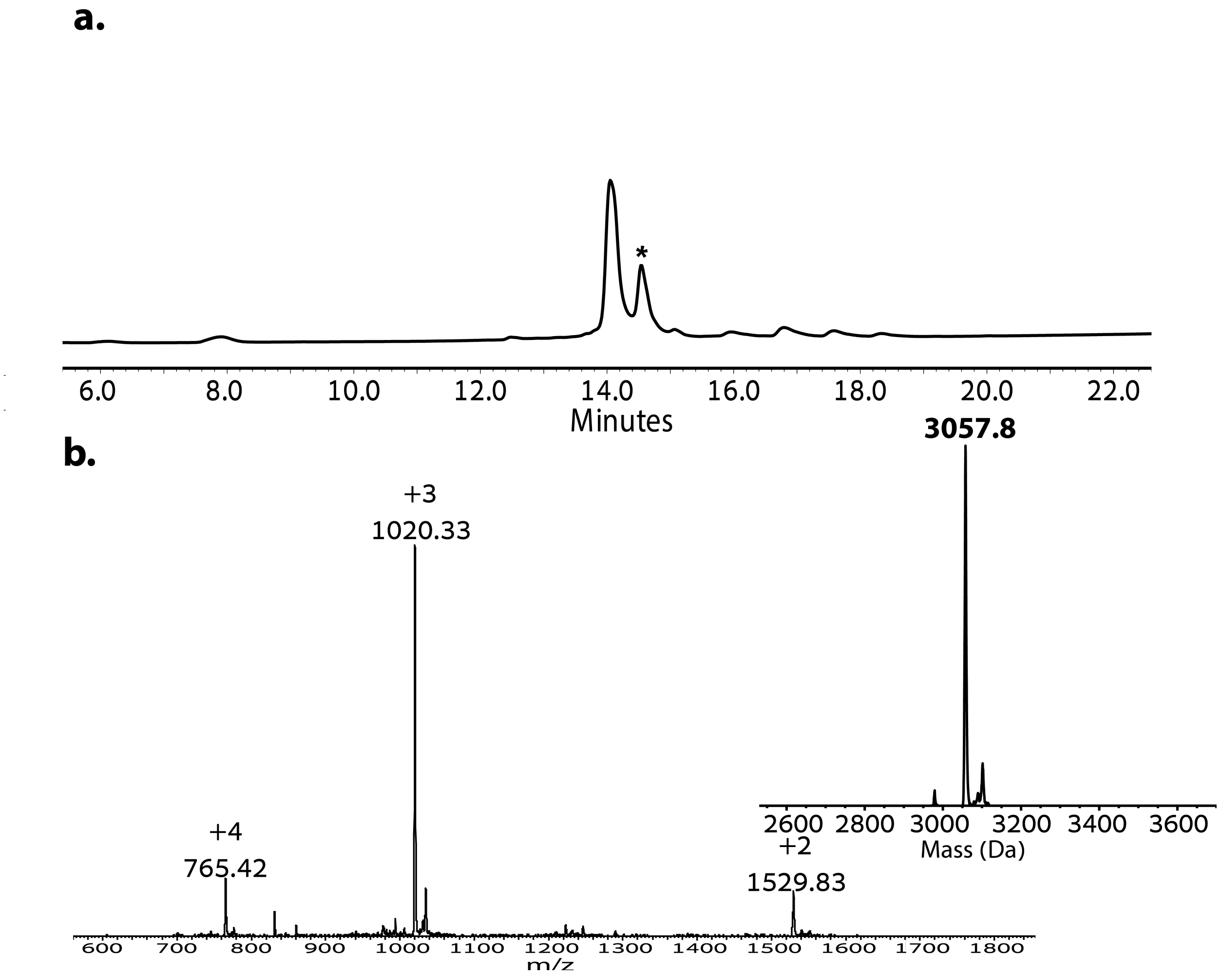

Figure S1. Characterization of the SELENOF(141-165)(A141U) segment. **a**. HPLC analysis (220 nm); **b**. corresponding ESI-MS, with its deconvoluted mass (inset) (obs. 3057.8± 0.3 Da, calc. 3058.3 Da). * corresponds to the dimer form of SELENOF(141-165)(A141U).

4.3.3. Synthesis of SELENOF(96-140)(Sec96FmocSez)-COSR and SELENOF(96-140)(Sec 96Sez)-COSR

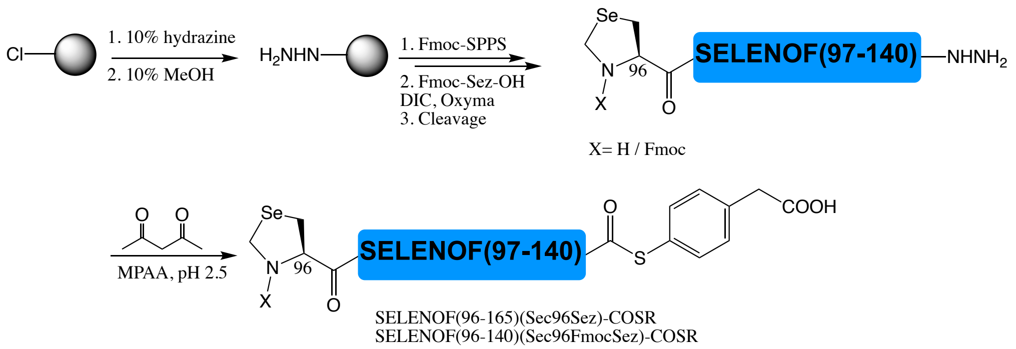

Scheme S2. Chemical synthesis of SELENOF(96-140)(Sec96FmocSez)-COSR and SELENOF(96-140)(Sec96Sez)-COSR by SPPS

The synthesis of SELENOF(96-140)(Sec96FmocSez)-COSR, was carried out on 2-chlorotrityl chloride resin, on a 0.25 mmol scale (0.4 mmol/g) was swelled in DMF and DCM for 1 h and treated twice with freshly prepared 10% hydrazine in DMF for 30 min and decanted.[4] The resin was washed well with DMF and then treated with 10% MeOH in DMF for 30 min. The hydrazide functionalized 2-chlorotrityl chloride resin was used for standard Fmoc-SPPS where the coupling of the amino acids held on an automated synthesizer. Fmoc-Sez-OH was manually coupled for 2-3 h (1.5 equiv Fmoc-Sez-OH activated on ice for 5 min using 3.0 equiv OxymaPure and 2.9 equiv DIC).[1] The cleavage was done by using TFA:TIPS:water (94:3:3) cocktail for 3-4 h.

The conversion to thioester was done by dissolving the peptide in phosphate buffer (200 mM, 6 M Gn·HCl, pH ~2.5) and treated with 50 equiv of acetylacetone (acac) and 100 equiv of MPAA for 3 h at room temperature.[5] Purification by RP-HPLC (C4 column) yielded 27% of pure peptide (Scheme S2, Figure S3).

Using the same synthesis described above SELENOF(96-140)(Sec96Sez)-COSR was prepared by coupling Boc-Sez-OH instead of Fmoc-Sez-OH, with the same coupling conditions (Scheme S2, Figure S2).

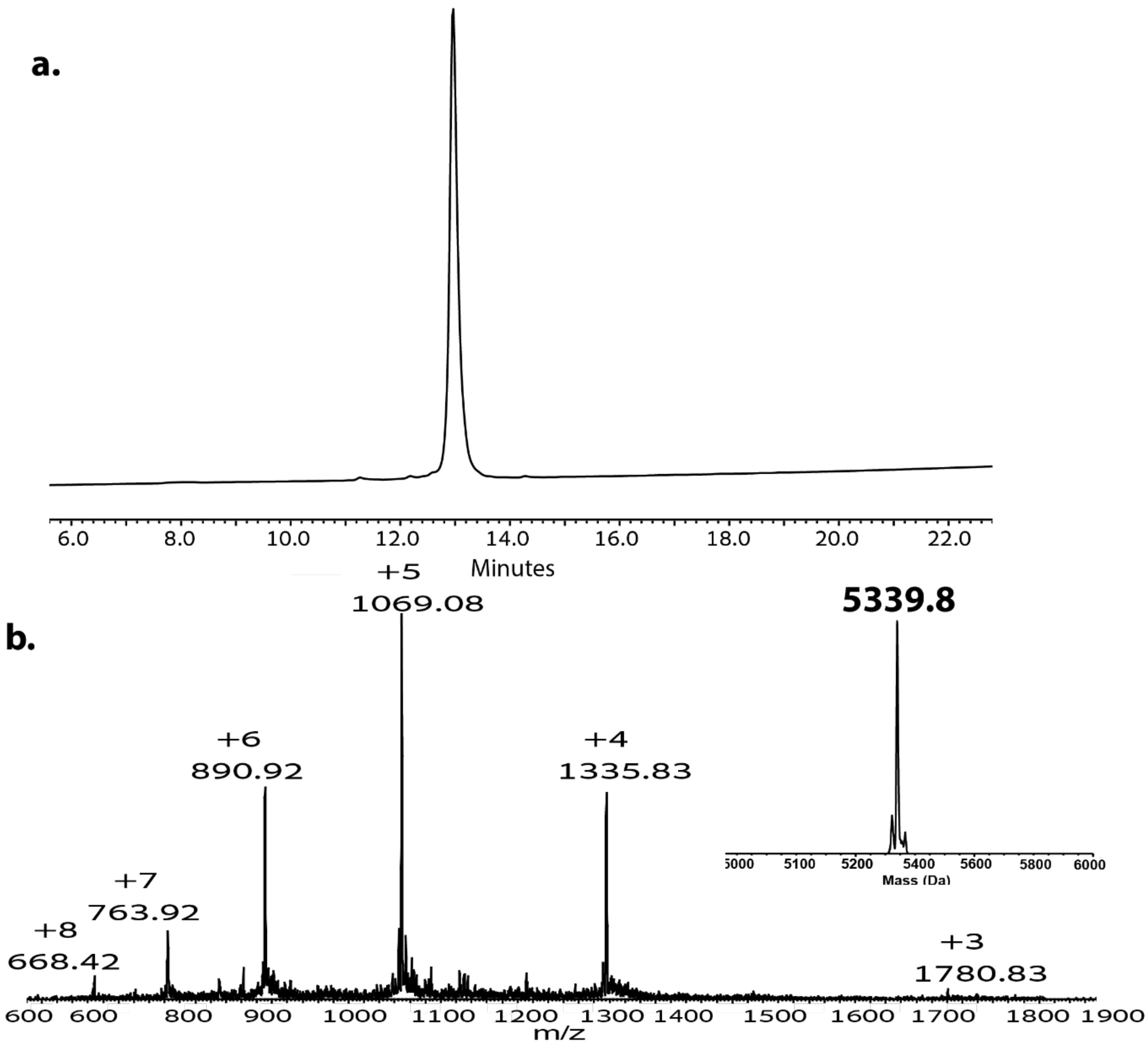

Figure S2. Characterization of the SELENOF(96-140)(Sec96Sez)-COSR segment. **a**. Analytical HPLC analysis (220 nm); **b**. The corresponding ESI-MS, with its deconvoluted mass (inset) (obs. 5339.8 ± 0.5 Da, calc. 5338.8 Da).

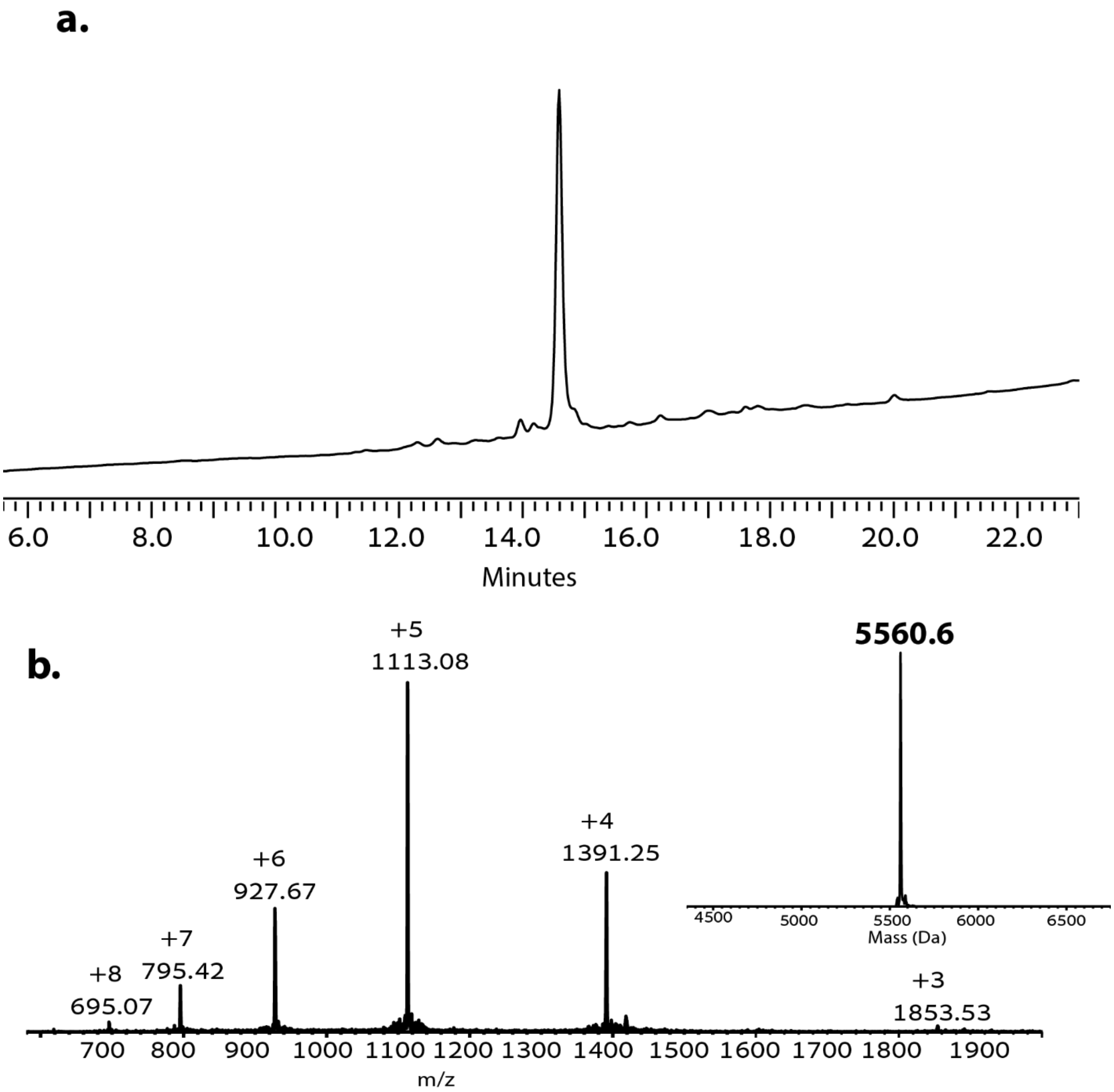

Figure S3. Characterization of the SELENOF(96-140)(Sec96FmocSez)-COSR segment. **a**. Analytical HPLC analysis (220 nm); **b**. The corresponding ESI-MS, with its deconvoluted mass (inset) (obs. 5560.60 ± 0.20 Da, calc. 5561.9 Da).

4.3.4. Synthesis of SELENOF(89-95)-COSR

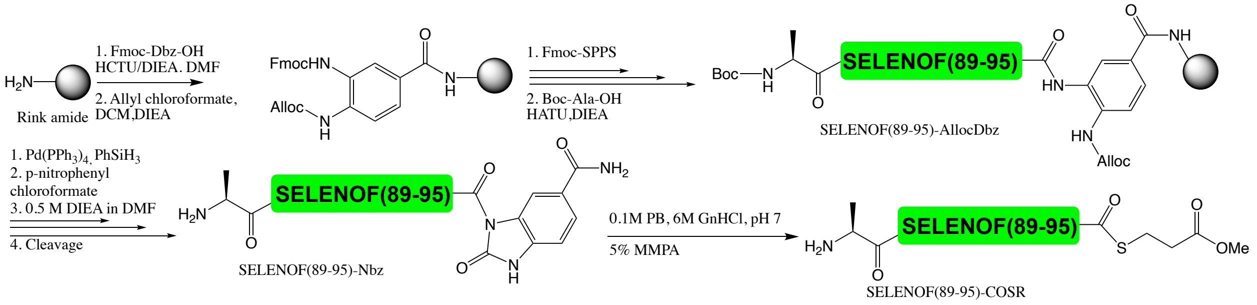

Scheme S3. Chemical synthesis of SELENOF(89-95)-COSR by SPPS

Since the C-terminal residue is Gly, the synthesis of SELENOF(89-95)-COSR was carried out manually on 0.25 mmol scale on the functionalized Fmoc-Dbz(Alloc)-resin.[6] Mono-Fmoc-3,4-diaminobenzoic acid (Fmoc-Dbz-OH, 3 equiv) activated with HCTU (3 equiv/DIEA 6 equiv) in DMF was doubly coupled manually to TentaGel® R RAM resin (0.19 mmol/g, 0.25 mmol scale) for 2 h.

Alloc protection: The Dbz functionalized resin was washed with DMF and DCM, and 0.35 M of allylchloroformate and 1 equiv of DIEA in DCM were added and shaken for 24 h at room temperature.[7]
Manual standard Fmoc-SPPS was preformed, including a final coupling of Boc-Ala-OH (3 equiv AA activated with 3.0 equiv HATU and 2.9 equiv DIEA) to yield the full-length sequence.

Alloc deprotection: After peptide synthesis is completed, the alloc protected peptide-resin was washed and swollen in DCM for 30 min and sparged very well with Ar, and 20 equiv of $PhSiH_3$ and 0.35 equiv of $Pd(PPh_3)_4$ in 5 mL DCM was added and shaken for 4 h.[7] Following deprotection the washing steps were preformed using 0.5 M DIEA in DMF, 20 mM sodium diethyldithiocarbamate trihydrate in DMF, DMF and DCM. Each washing step was repeated three times.[7]

Nbz formation: The peptide-resin was washed with DCM and a solution of *p*-nitrophenyl chloroformate (5 equiv) in DCM was added and mixed for 1 h, washed with DCM (3 × 5 mL) and DMF (3 × 5 mL). This step was repeated two more times. To complete the Nbz formation, 5 mL of 0.5 M DIEA in DMF was added and shaken for additional 30 min. When this step was repeated

twice, the resin was washed with DMF and DCM.[8] Using the standard cleavage cocktail (TFA, TIPS, water) (95%, 2.5%, 2.5%) the on-resin peptide-Nbz form was cleaved and lyophilized.

Thioesterification: the crude peptide-Nbz (3 mM) was dissolved in phosphate buffer (200 mM, 6 M Gn·HCl, pH ~7) and treated with methyl 3-mercaptopropionate (MMP) (5% v/v) for 4 h, at room temp.[8] Purification by RP-HPLC (C18 column) yielded ~ 43 % of pure peptide (Scheme S3, Figure S4).

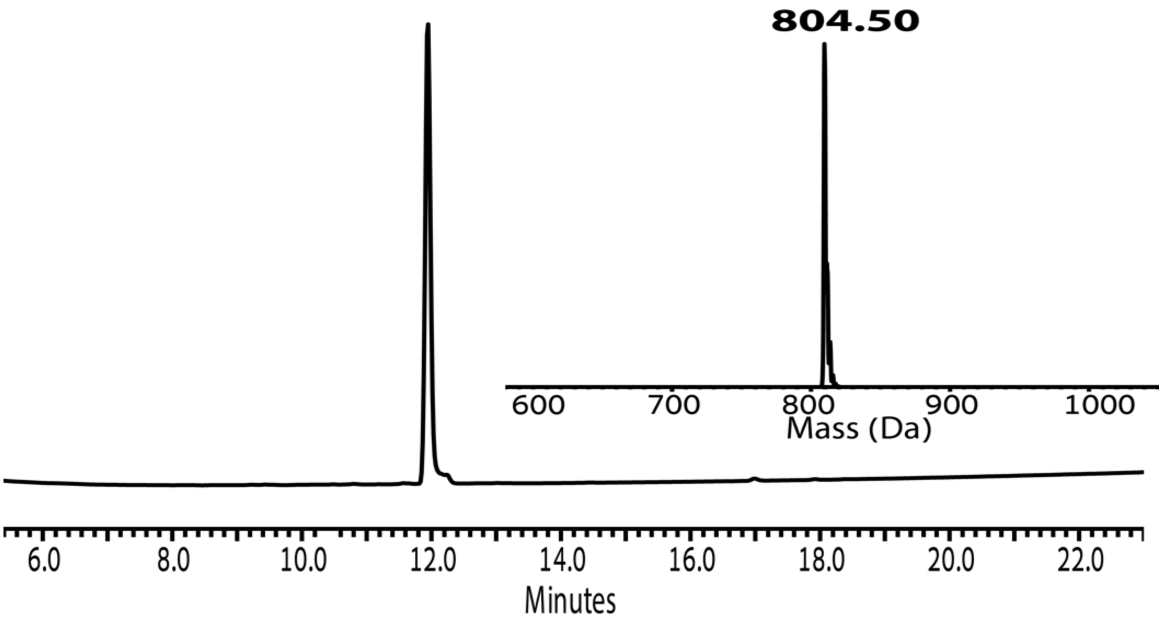

Figure S4. Characterization of the SELENOF(89-95)-COSR segment. Analytical HPLC analysis (220 nm); and the corresponding ESI-MS, with its deconvoluted mass (inset) (obs. average 804.5 Da, calc. 805.4 Da).

4.4. Native chemical ligation (NCL), Deselenization and deprotection reactions

4.4.1. 1st ligation reaction

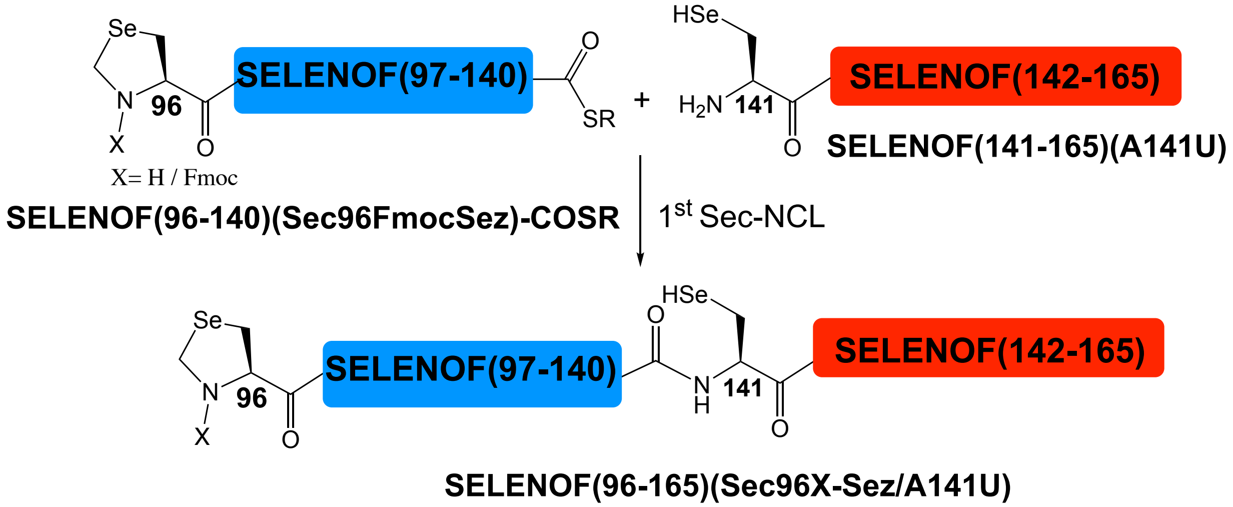

Scheme S4. 1st NCL between SELENOF(96-140)(Sec96Sez)-COSR (X = H) or SELENOF(96-140)(Sec96FmocSez)-COSR (X= Fmoc) with SELENOF(141-165)(A141U).

Ligation of SELENOF(96-165)(Sec96Sez/A141U)

The ligation between SELENOF(96-140)(Sec96Sez)-COSR (5.0 mg, 0.94 µmol, ~1 mM) and SELENOF(141-165)(A141U) (~4.3 mg, 1.4 µmol, ~1 mM) in 1 mL of argon degassed phosphate buffer (200 mM PB, 6 M Gn·HCl, pH 7) at 37 °C for 18 h in the presence of 0.05 M TCEP and 0.1 M of sodium ascorbate to yield SELENOF(96-165)(Sec96Sez/A141U) (Figure S5). The reaction was purified by semi-prep HPLC (C4 column).

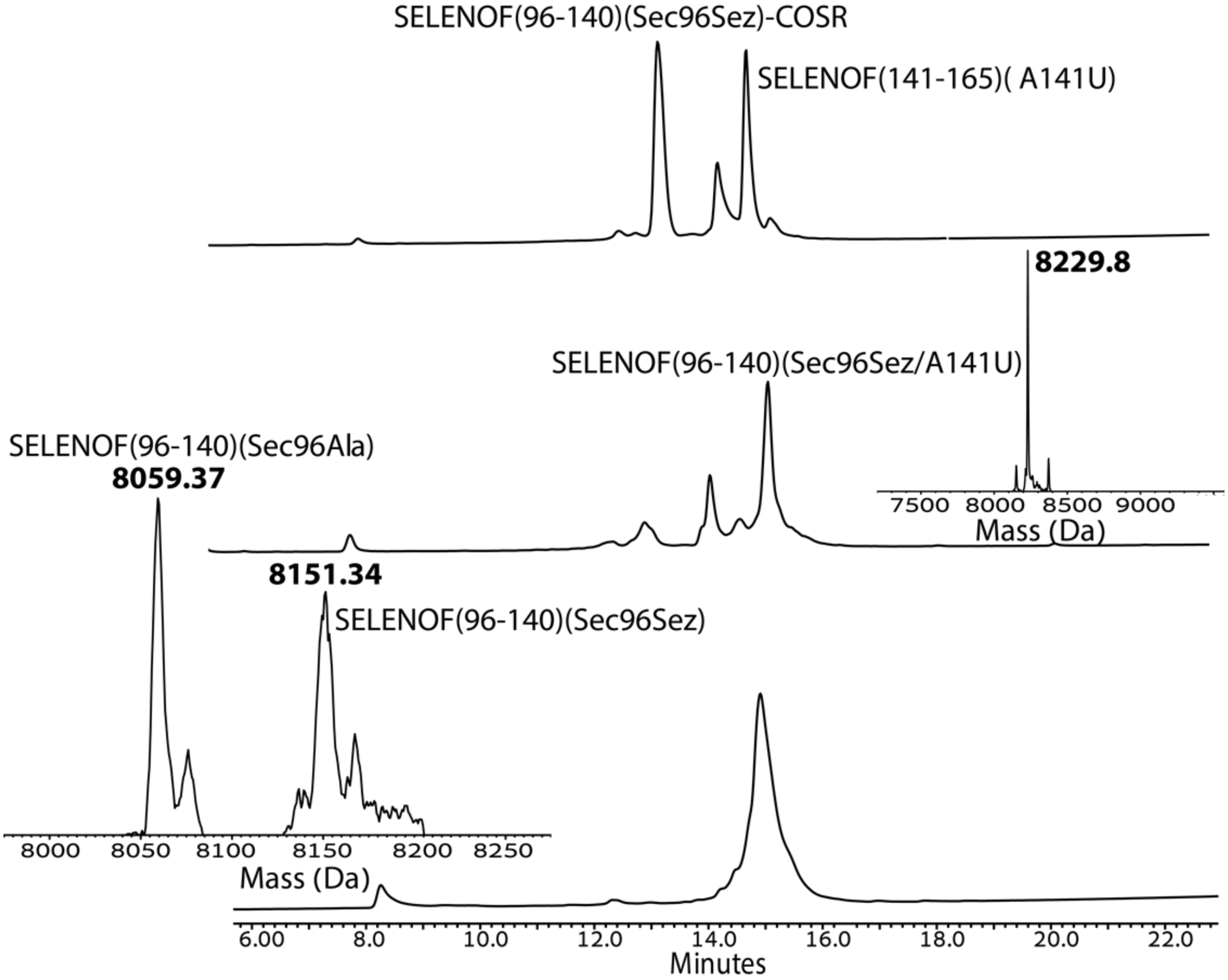

Figure S5. 1st NCL reaction between SELENOF(96-140)(Sec96Sez)-COSR and SELENOF(141-165)(A141U) followed by purification with semi-prep HPLC (C4 column). The purified product was subjected to the deselenization reaction with TCEP at pH ~5, and under anaerobic conditions. The deconvoluted mass of SELENOF(96-165)(Sec96Sez/A141U)) (inset) (obs. average 8229.8±0.9 Da, calc. 8230.3 Da), for SELENOF(96-165)(Sec96Sez) is (inset) (obs. average 8151.3±0.5 Da, calc. 8151.3 Da) and for SELENOF(96-165)(U96A) is (inset) (obs. average 8059.4±0.6 Da, calc. 8060.3 Da). It is clearly observed that undesired deselenization at position 96 is a major side product.

Ligation of SELENOF(96-165)(Sec96FmocSez/A141U)

The peptide SELENOF(96-140)(Sec96FmocSez)-COSR (11.0 mg, 2.0 μmol, 5.0 mM) was incubated together with SELENOF(141-165)(A141U) peptide (~8.0 mg, 2.6 μmol, ~5.2 mM) in 0.4 mL of argon degassed phosphate buffer (200 mM PB, 6 M Gn·HCl, pH 7) at 37 °C. To accelerate the NCL reaction at the C-terminal Ile, 4 equiv of TCEP were added in four portions each within a 2 h interval. The reaction progress was followed by analytical HPLC (C4 column) and completed in 10 h. (Scheme S4, Figure S6 and Figure 2c in the main manuscript).

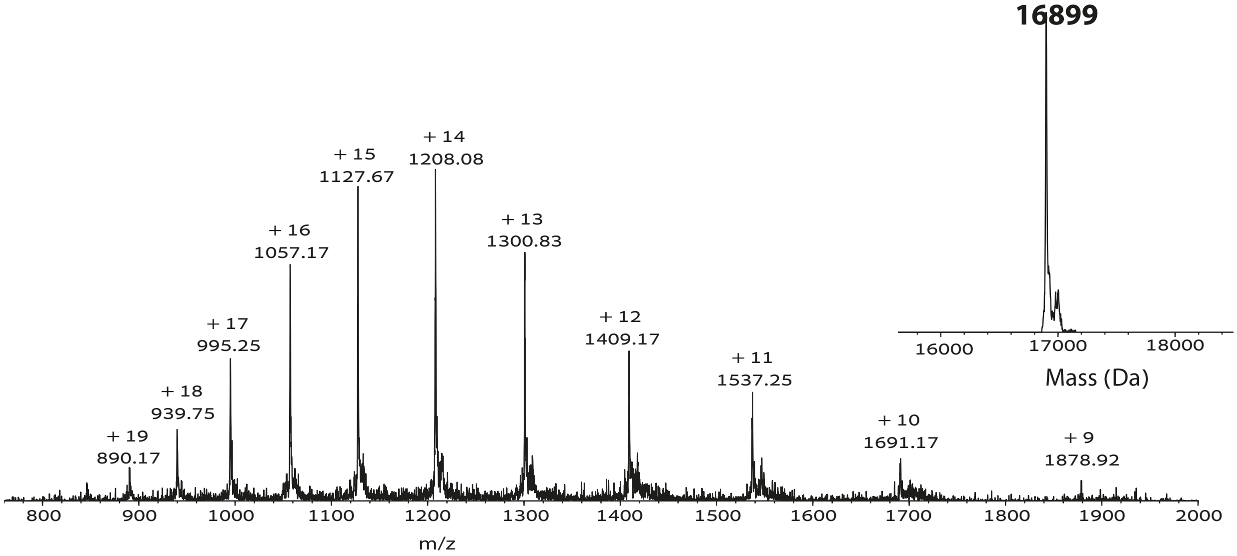

Figure S6. ESI-MS characterization of the SELENOF(96-165)(Sec96FmocSez/A141U) segment in a dimer form, with its deconvoluted mass (inset) (obs. average 16899.0 ± 2.3 Da, calc. 16903.0 Da).

4.4.2. Deselenization reactions

4.4.2.1. Testing the epimerization during the removal of Tfa group from Sez

The model peptide Tfa-Sez-LYRAG-NH_2 (0.9 mg) was dissolved in 360 μL degassed phosphates buffer (0.2 M, with 8 M guanidine, pH 7.08) to a final concentration of 3 mM. Using 10 M NaOH the pH was raised to 11-12 and the Tfa protecting group was removed completely within 1 h. Next, the pH of the reaction mixture was lowered to 5-6 using 5 M HCl, and 3 equiv of $CuCl_2$ and 5

equiv of $MeONH_2$ were added to the solution. As Sez opening occurred the reaction was purged with argon and transferred to anaerobic chamber. The Deselenization reaction preformed in the presence of 16.2 mg TCEP (60 equiv) and 10.4 mg VA-044 (30 equiv) and kept for 18 h to yield a mixture of (*l*)-**A**LAYRAG-NH_2 and (*d*)-**A**LAYRAG-NH_2. The results of this experiment are represented in Figure S7, where we can clearly observe the epimerization.

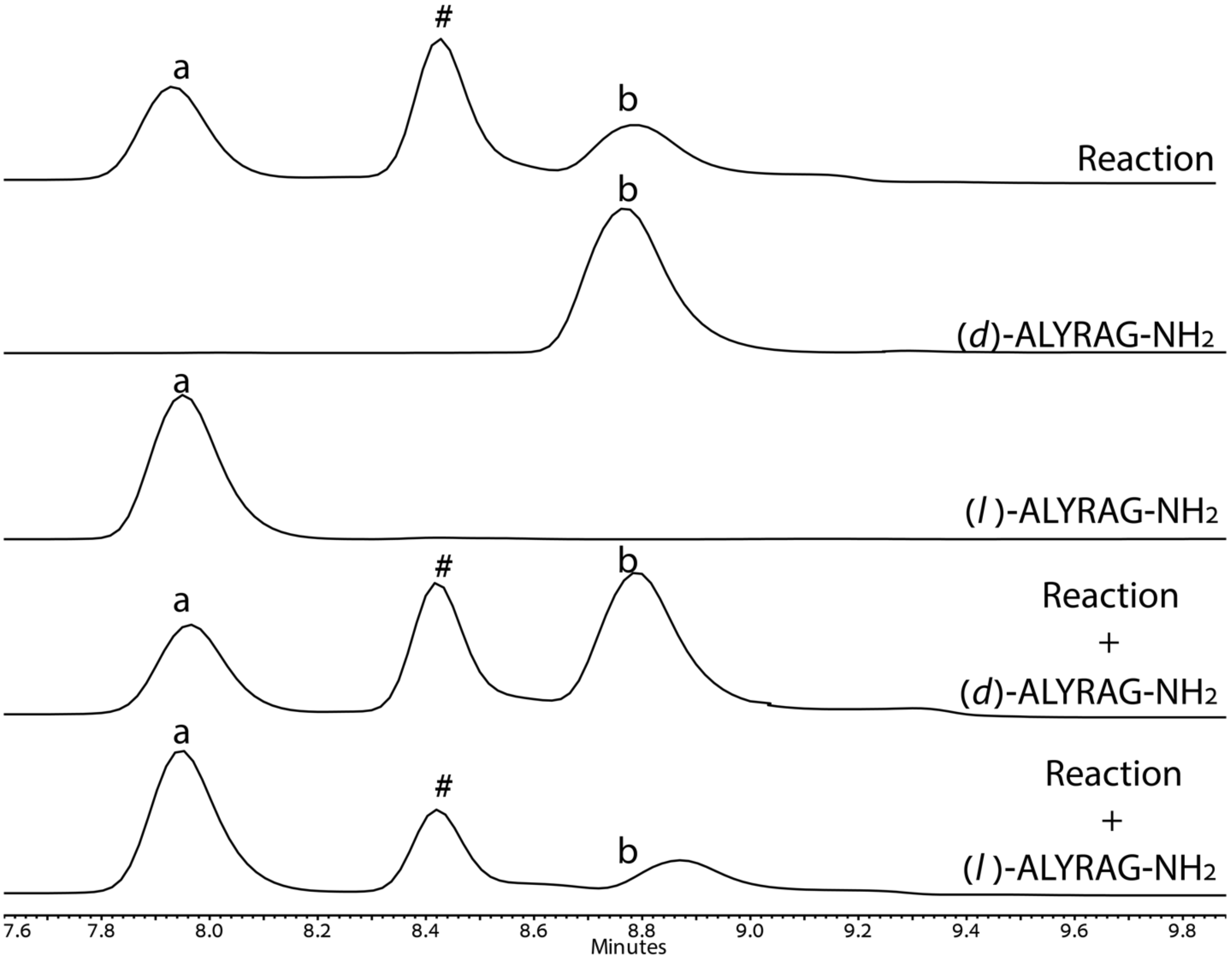

Figure S7. Epimerization study for Tfa-protected Sez containing model peptide, Tfa-Sez-LYRAG-NH_2. Deprotection was done in phosphate buffer at pH 11, followed by a Sez opening using $CuCl_2$ and $MeONH_2$ at pH 4-5. Deselenization at pH 5 using 60 equiv of TCEP. # denotes to TCEP=Se adduct, **a** is (*l*)-**A**LYRAG-NH_2 and **b** is (*d*)-**A**LYRAG-NH_2.

4.4.2.2.Deselenization of SELENOF(96-165)(Sec96FmocSez/A141U)

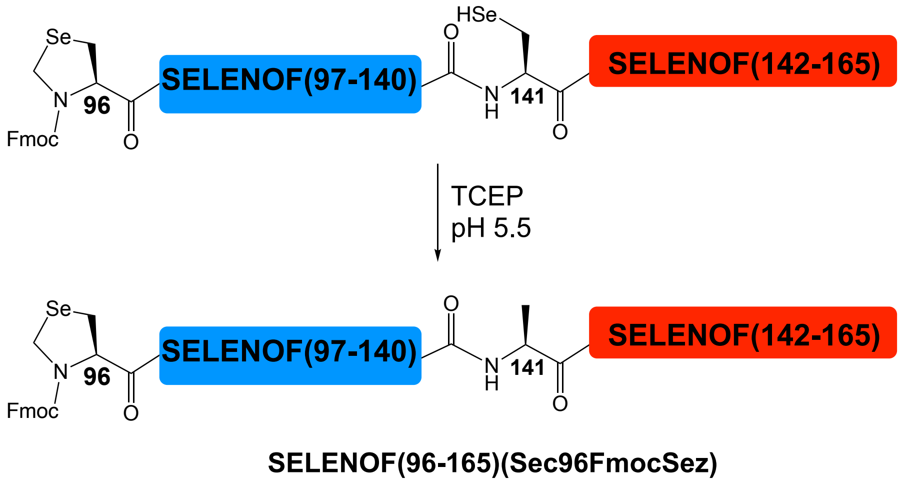

Scheme S5. Deselenization reaction of SELENOF(96-165)(Sec96FmocSez/A141U)

Following ligation SELENOF(96-165)(Sec96FmocSez/A141U) was directly deselenized in the in the presence of 50 equiv of TCEP at pH 5.5 at 37 °C.[9,10] Argon was purged into the reaction for 5-10 min, and it was held under anaerobic conditions. Within 13 h the reaction completed to yield the desired product SELENOF(96-165)(Sec96FmocSez) (Scheme S5, Figure S8 and Figure 2c in the main manuscript).

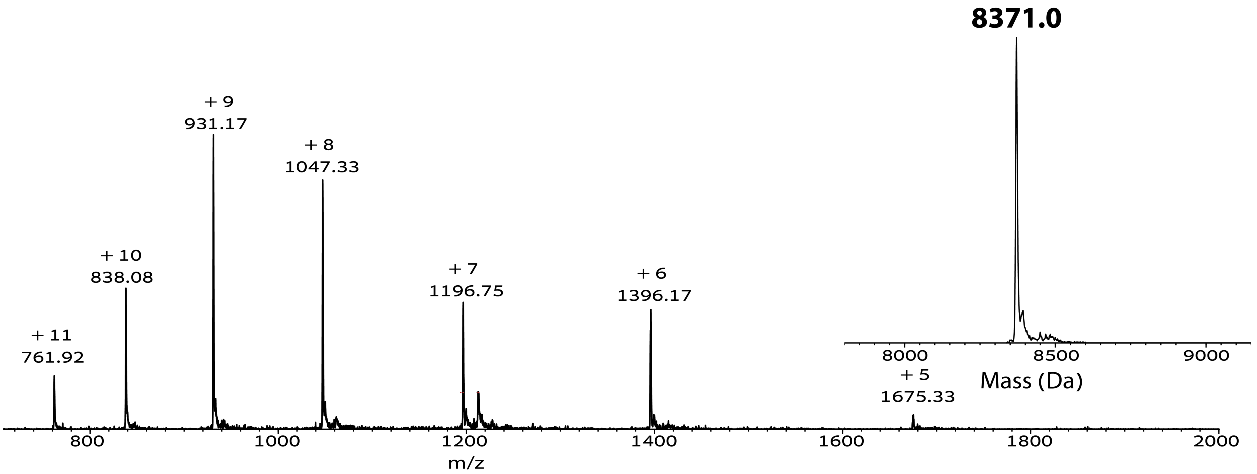

Figure S8. ESI-MS characterization of the SELENOF(96-165)(Sec96FmocSez) segment, with its deconvoluted mass (inset) (obs. average 8371.0 ± 0.5 Da, calc. 8373.5 Da).

4.4.3. Fmoc deprotection and Sez opening

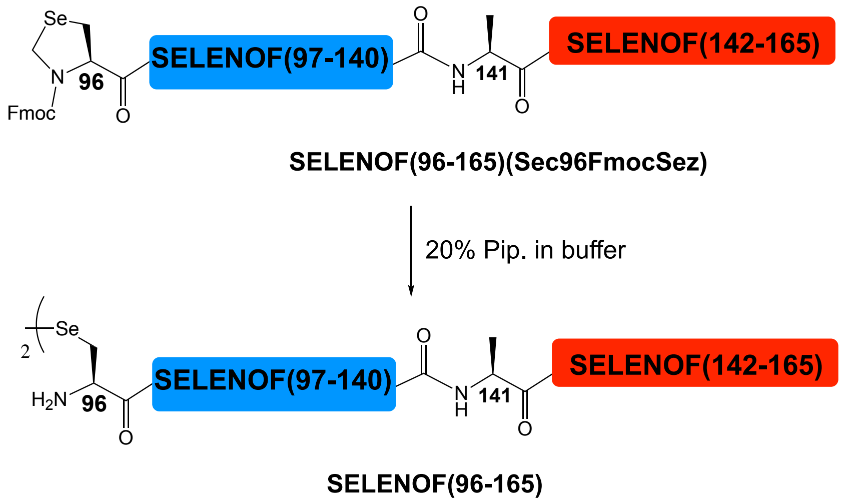

Scheme S6. One-pot reaction of Fmoc deprotection and Sez opening

Fmoc deprotection of SELENOF(96-165)(Sec96FmocSez) was achieved by treating the peptide with 20% piperidine[11] in the phosphate buffer at pH 10. After 6 h the reaction was completed to offer one-pot Fmoc deprotection and Sez opening. To avoid undesired deselenization at position 96, 100 mM of sodium ascorbate was added to the reaction mixture. SELENOF(96-165) was analyzed as a mixture of dimer and monomer form (Scheme S6, Figure S9 and Figure 2c in the main manuscript).

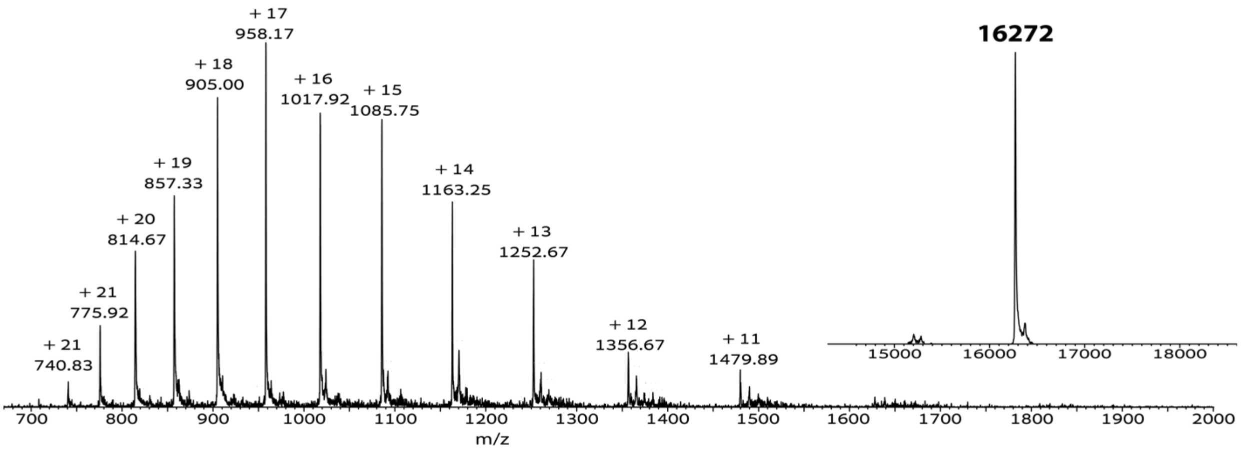

Figure S9. ESI-MS characterization of the SELENOF(96-165) segment in a dimer form, with its deconvoluted mass (inset) (obs. average 16272.0 ± 1.9 Da, calc. 16271.6 Da).

4.4.4. 2nd ligation reaction

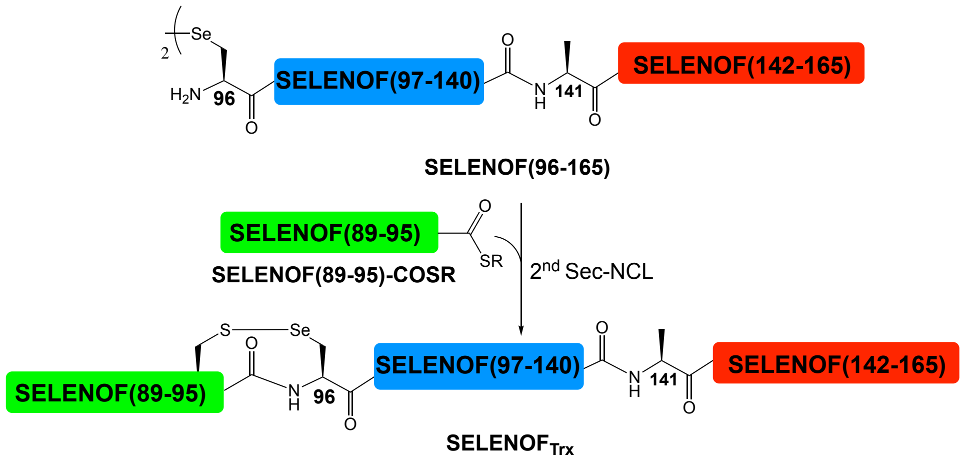

Scheme S7. 2nd NCL between SELENOF(96-165) and SELENOF(89-95)-COSR

Without any purification step, the second ligation was preformed directly after the deprotections steps, where SELENOF(89-95)-COSR (1.6 mg, 2 μmol) was added to the reaction mixture together with 100 equiv of MPAA and the pH was adjusted back to ~7. Within 3 h the reaction was completed and purified by semi prep (C4 column) to yield 22.6% (4 mg) of full-length protein (Scheme S7, Figure S10 and Figure 2c in the main manuscript).

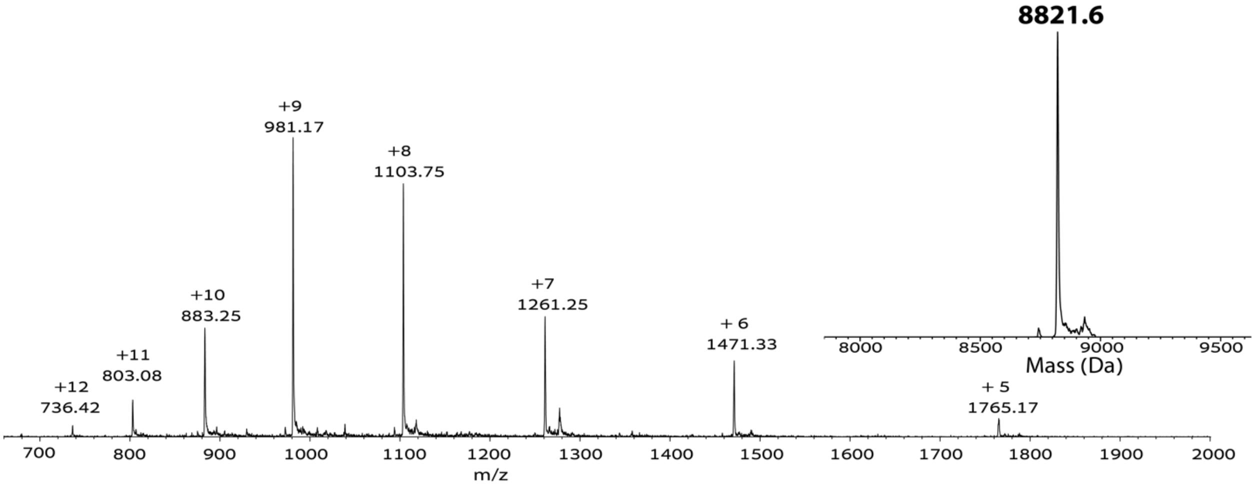

Figure S10. ESI-MS characterization of the SELENOF(89-165), with its deconvoluted mass (inset) (obs. average 8821.6 ± 1.2 Da, calc. 8822.11 Da).

4.5. Circular Dichroism (CD)

Sample preparations- 0.5 mg of $SELENOF_{Trx}$ was dissolved in 300 µL of the folding buffer (10 mM phosphate, 150 mM NaCl, pH 9). The solution was kept for 2 h to allow the folding, and the sample was centrifuged at 5000 rpm for 3 min, the dissolved solution was taken, and the concentration was determined by NanoDrop UV-Vis spectrophotometer (using theoretical $\varepsilon_{280\ nm} = 6990\ M^{-1} \cdot cm^{-1}$). The final concentration of the protein was calculated to be 34.3 µM. Following folding the secondary structure feature of the synthetic human $SELENOF_{Trx}$ was analyzed in the far-UV CD spectroscopy (200 to 250 nm). Spectra were recorded on J-810 spectropolarimeter (Jasco), using a quartz cuvette with a path length of 0.1 cm, and obtained by averaging 5 wavelength scans in 0.1 nm steps, with a signal averaging time of 2 s and a bandwidth of 1 nm (Figure S11).

$$\theta_{MER} = \frac{\theta(mdeg)}{10 \times l(cm) \times c(M) \times N(\#\ amide\ bond)}$$

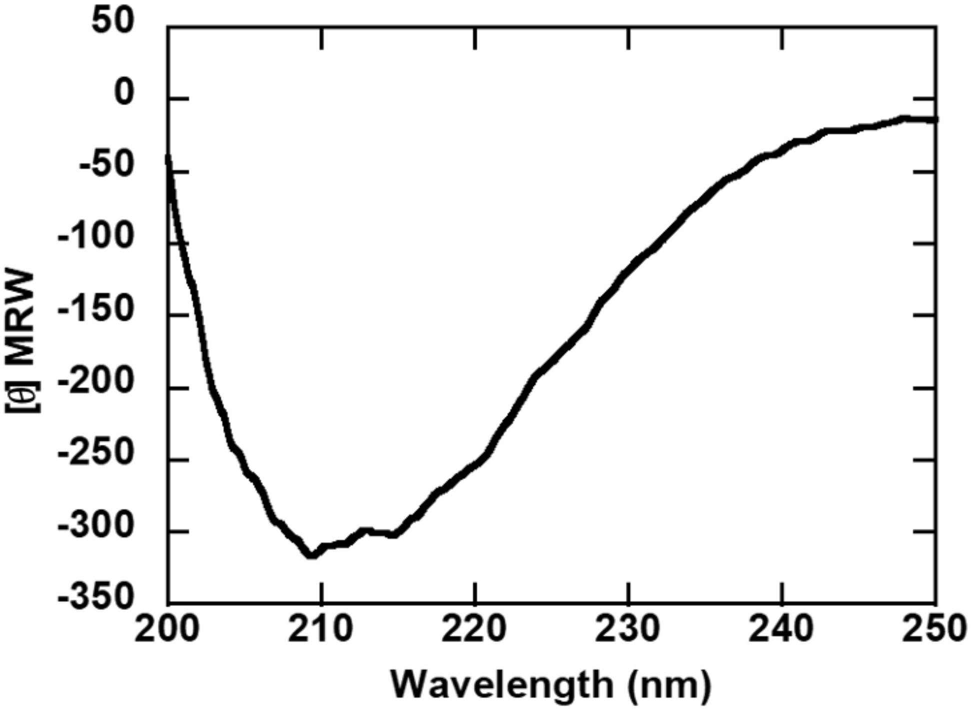

Figure S11. CD spectra of $SELENOF_{Trx}$.

4.6. Redox potential determination

The redox potential of the Trx-like domain of SELENOF was determined using the method of protein-protein redox equilibria.[12] Equimolar amounts of oxidized $SELENOF_{Trx}$ and freshly reduced *E.coli* Trx were used and the reaction was followed by HPLC. First, the *E. coli* Trx was dissolved in 200 μL of argon-degassed phosphate buffer (100 mM, 1 mM EDTA, pH 7.01) and reduced with 2 equiv of TCEP, followed by centrifugation-dialysis (AmiconUltra, cutoff 5000 Da, Millipore Crop., Bedford, MA) by degassed phosphate buffer (5 × 2 mL). $SELENOF_{Trx}$ was allowed to fold similarly as mentioned (in SI 4.5). The concentration of each protein was determined by NanoDrop UV-Vis spectrophotometer, using the following values: *E. coli.* Trx (ε_{280nm} = 13700 cm^{-1} M^{-1}); $SELENOF_{Trx}$ (ε_{280nm}= 6990 cm^{-1} M^{-1}).[13] The redox equilibration between the two proteins (final concentration of both proteins is 15.4 μM) was carried under anaerobic conditions where both proteins were incubated together in the assay buffer (100 mM phosphate, 1 mM EDTA, pH 7.01). The reaction was stopped after the desired time interval (from 30 sec to 8 min) by taking aliquots from the reaction, quenched with 2 M HCl and immediately injecting into the HPLC. [12,14]

Although the reaction was done under anaerobic conditions and in argon purged solutions still we observed some oxidation of Trx by trace oxygen, which was also taken into account where Trx was allowed to oxidize in the absence of $SELENOF_{Trx}$. At each time point the amount of oxidized Trx was calculated by subtracting the peak area of oxidized Trx in the control experiment from the peak area of Trx in the redox equilibria reaction. The oxidized and reduced form of Trx were separated by HPLC on a C18 column using a gradient of 35-70% (v/v) buffer B in 25 min at a flow rate of 1 mL/min, monitoring at 220 nm. The amount of oxidized and reduced form of Trx in the quenched mixture was determined by peak area integration. The percentage of the oxidized and reduced form of Trx were calculated at the final time point (8 min), when equilibrium was reached, and it is the average of three separate experiments. K_{eq} was calculated based on the concentration of reduced and oxidized Trx at equilibrium (eq 1) and introduced into Nernst equation (eq 2) to calculate the difference in redox potential, ΔE. n is the electron number transferred (here n = 2), F is Farady's constant (23.04 kcal mol^{-1} V^{-1}), R is the gas constant (1.987 cal K mol), T is the absolute temperature (298 K) and E_0 is the redox potential value of *E. coli* Trx (-270 mV) (See Figure 3a, b and c in the manuscript).[13]

$$\textbf{eq 1} \qquad K_{eq}=\frac{[SELENOF_{red}][Trx_{ox}]}{[SELENOF_{ox}][Trx_{red}]} = K_{eq}=\frac{[Trx_{ox}]^2}{[Trx_{red}]^2}, \text{ at equilibrium } [SELENOF_{red}] = [Trx_{ox}]$$

$$\textbf{eq 2} \qquad E = E_0 - \frac{RT}{nF}\ln K_{eq}$$

4.7. Turbidimetric assay

The reaction was carried in degassed 0.1 M phosphate buffer, 2 mM EDTA at pH 7 in a total volume of 300 µL. The assay mixture was prepared in the cuvette by the addition of insulin (final conc. 0.13 M) and $SELENOF_{Trx}$ (final conc, 7.8 µM). The reaction was started by adding DTT (final conc. 0.33 mM), mixed and scanned at 650 nm using 0.17 min recording on Thermo Scientific Evolution 201 UV-Visible spectrophotometer. The blank sample was also recorded in the absence of $SELENOF_{Trx}$ (See Figure 4 in the manuscript).[15]

The turbidimetric assay of insulin in the presence of *E. coli* Trx was held and recorded using the same conditions mentioned above.

4.8. Oxidative folding studies

The oxidative folding of both BPTI and hirudin was preformed according to the reported studies by Weissman and Kim for BPTI[17] and Chang *et al.*[16] for hirudin. All folding reactions were held under anaerobic conditions in an anaerobic chamber (Coy Laboratories Inc. O_2 sensor kept <5 ppm) with nitrogen and hydrogen atmosphere (95%:5%) in degassed Tris·HCl buffer (100 mM Tris·HCl, 200 mM NaCl, 1mM EDTA, pH 8.7).

BPTI folding Oxidized and reduced glutathione (GSSG, final concentration 0.2 mM and GSH final concentration 1 mM, respectively), and $SELENOF_{Trx}$ (final concentration 5 µM) were added to 30 µM of BPTI. At various time intervals, 80 µL aliquots were removed and quenched with 30 µL of 2 M HCl and stored at -20 °C before analysis by analytical HPLC. The reaction mixture was injected into Atlantis T3 column (3 µm 4.6 × 150 mm heated to 40°C) and eluted from the column by 10:90 to 26.5:73.5 gradient over 5 min (B:A), and increasing to 40:60 over 20 min. All chromatograms were monitored at a wavelength of 214 nm. (Figure S12a, b)

Hirudin folding Oxidized glutathione (5 equiv of GSSG, final concentration 150 µM) and $SELENOF_{Trx}$ (final concentration 5 µM) were added to 30 µM of hirudin. Similar to BPTI folding, at different time intervals, 80 µL aliquot was removed and quenched with 30 µL of 2 M HCl, and stored at -20°C before analysis by analytical HPLC. The reaction mixture was injected into Atlantis T3 column (3 µm 4.6 × 150 mm heated to 40°C) and eluted from the column by 15:85 to 22:78 gradient over 32 min (B:A), and reaching the initial gradient over 36 min . All chromatograms were monitored at a wavelength of 220 nm. (Figure S12c, d)

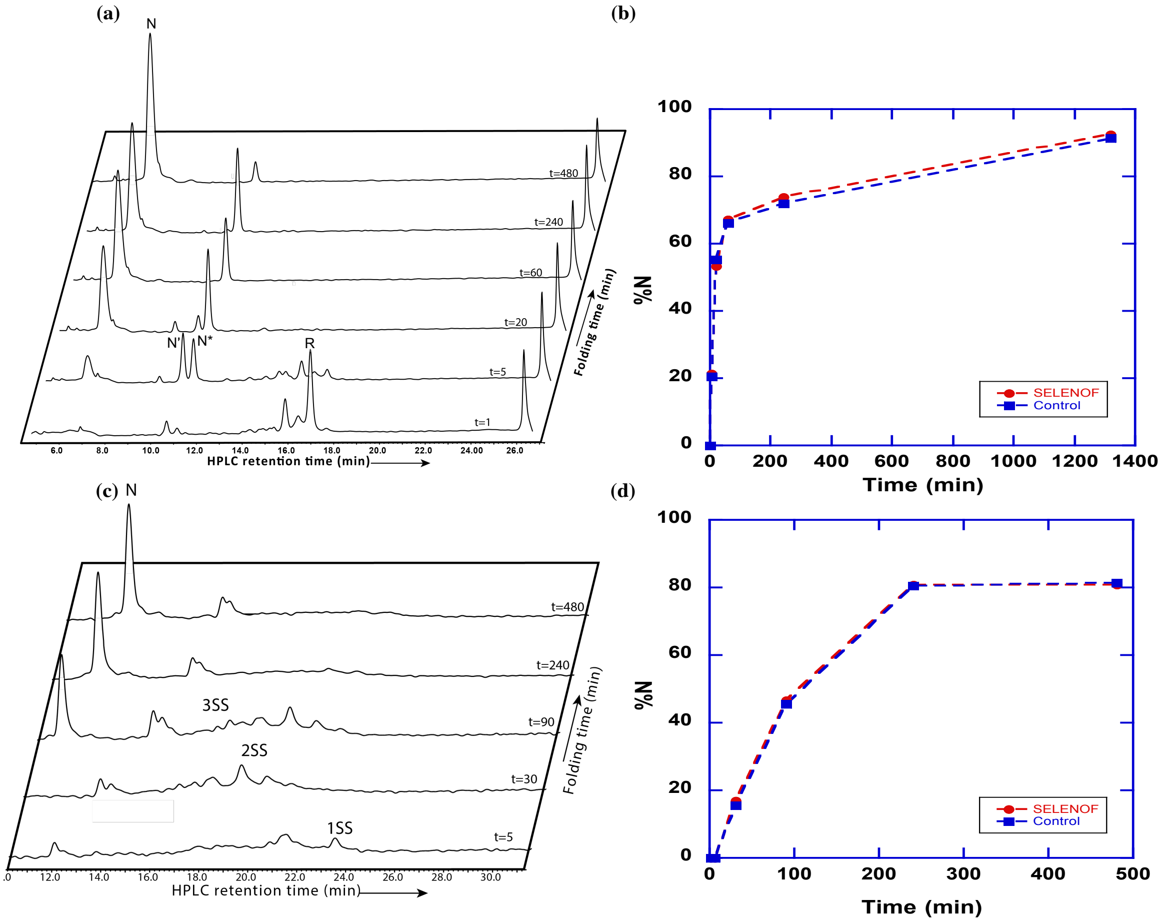

Figure S12. The oxidative folding analysis under anaerobic conditions and pH 8.7 of (**a**) 30 μM of BPTI in the presence of 1 mM GSH, 0.2 mM GSSG and 5 μM of $SELENOF_{Trx}$; and (**b**) Kinetic traces of the oxidative folding of BPTI in the presence (*red*) and the absence (*blue*) of $SELENOF_{Trx}$; (**c**) 30 μM of hirudin in the presence of 150 μM of GSSG and 5 μM of $SELENOF_{Trx}$; (**d**) Kinetic traces of the oxidative folding of hirudin in the presence (*red*) and the absence (*blue*) of $SELENOF_{Trx}$.

4.9. HR-MS

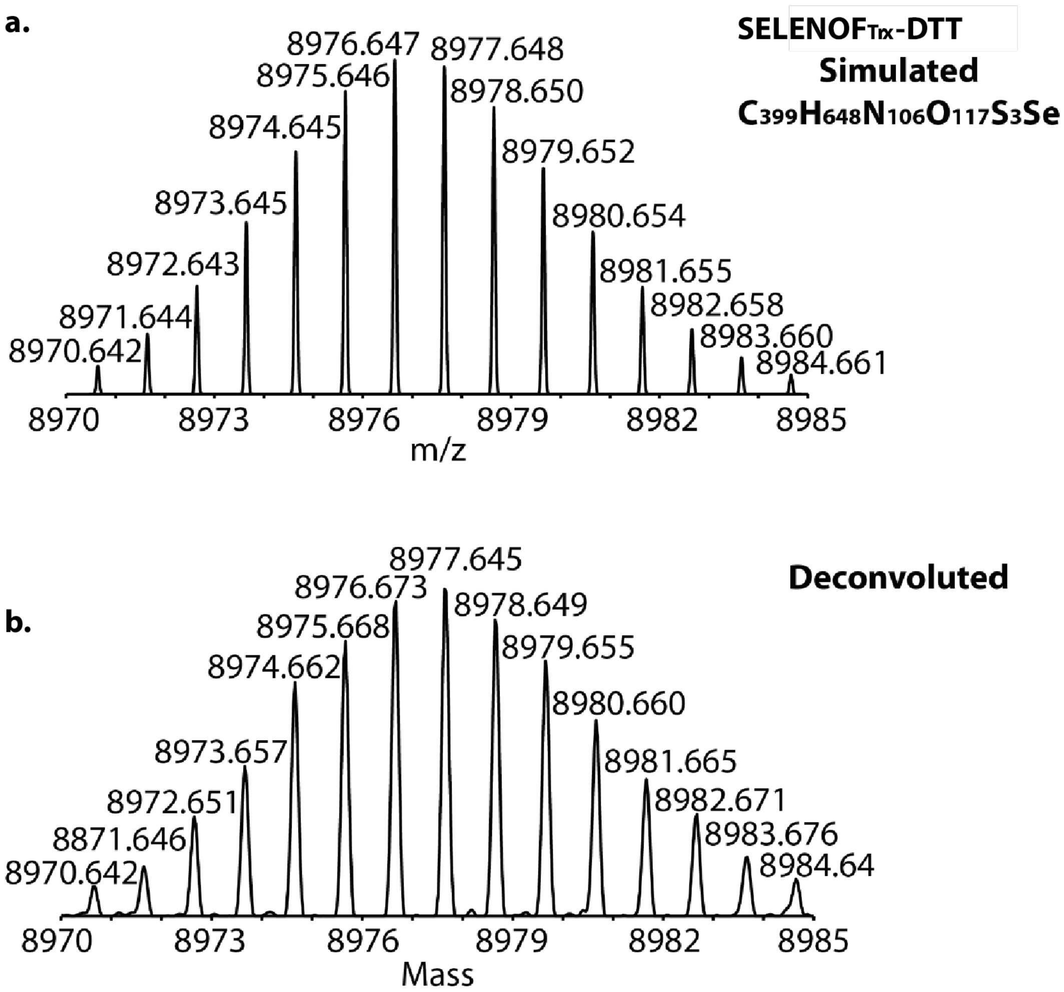

Figure S13. HR-MS analysis of SELENOF$_{Trx}$-DTT adduct. **a**. The simulated HR-MS of SELENOF$_{Trx}$ adduct, with the chemical formula $C_{399}H_{648}N_{106}O_{117}S_3Se$ shown. **b**. The deconvoluted HR-MS of SELENOF$_{Trx}$.

5. NMR Results

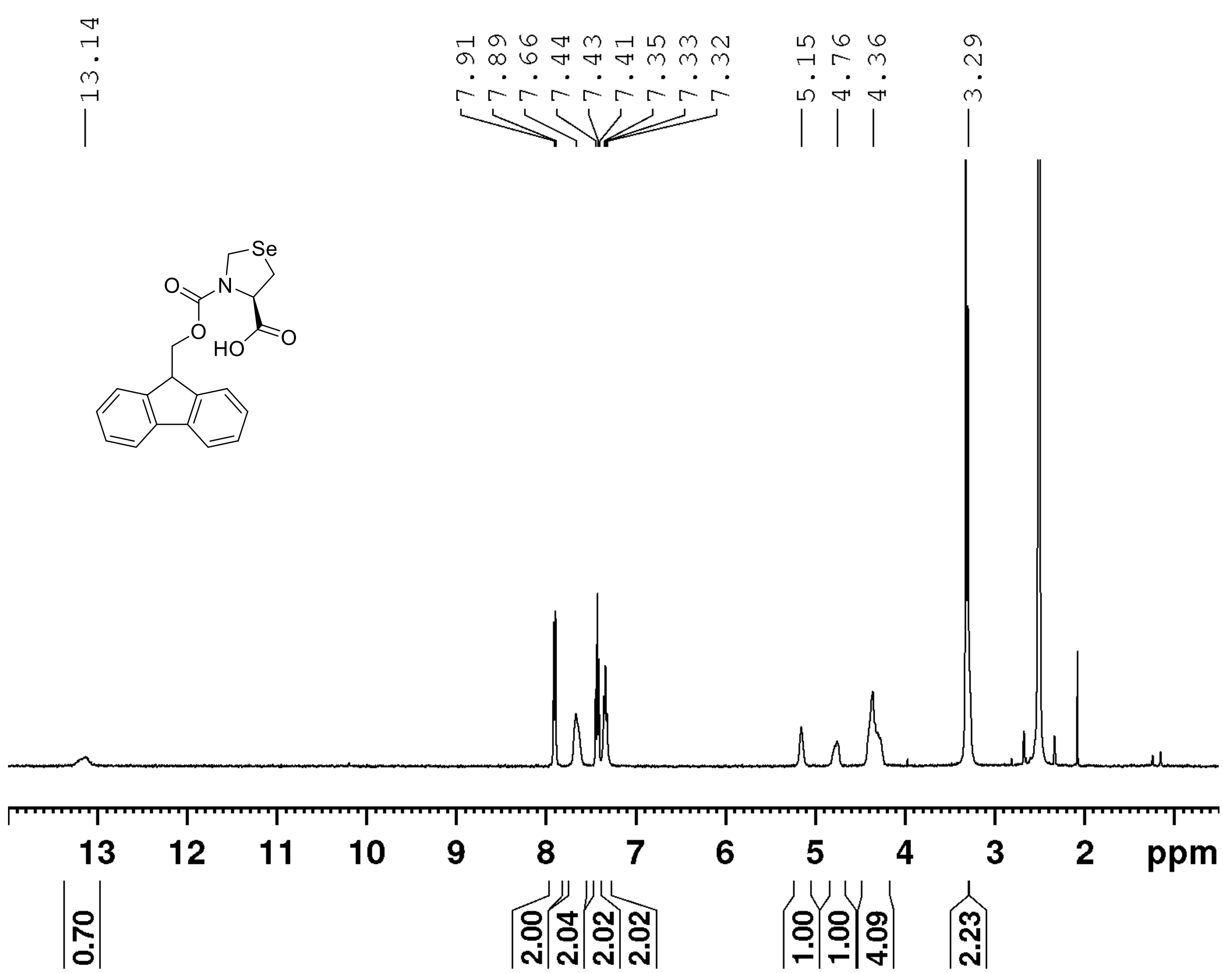

Figure S14. ^{1}H-NMR of Fmoc-Sez-OH

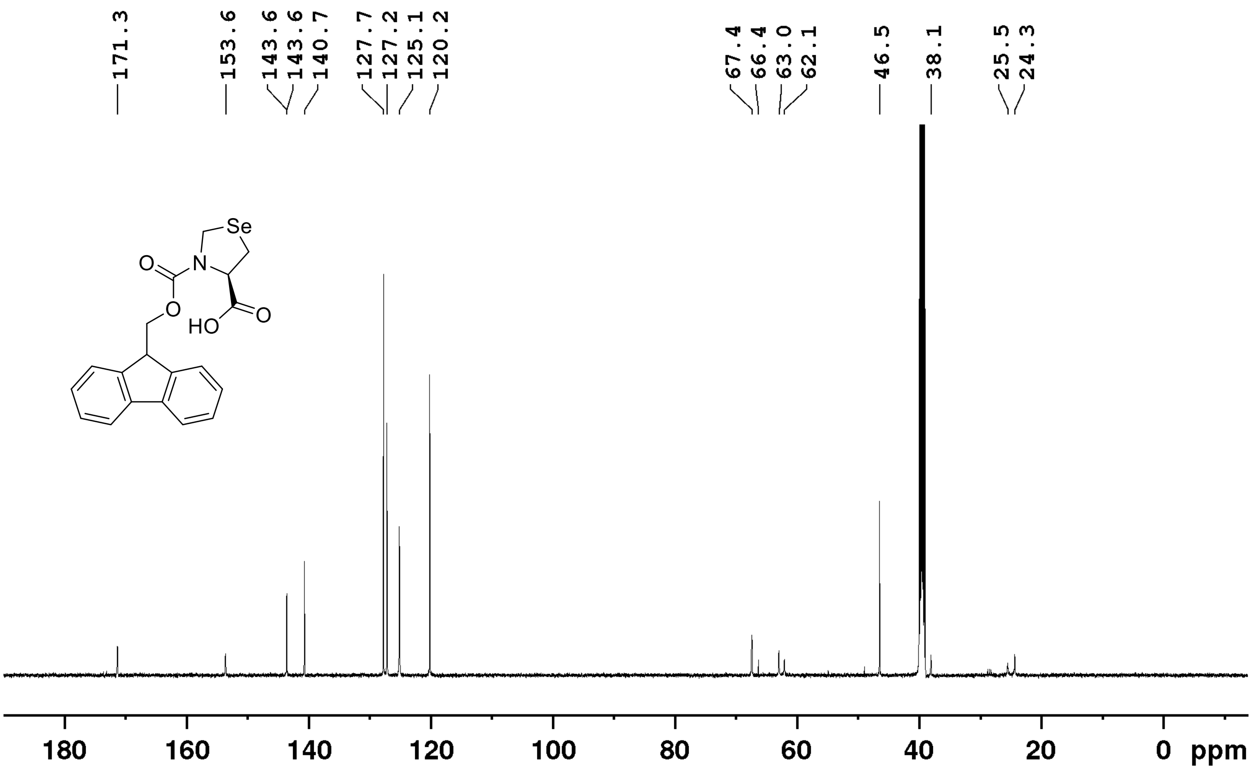

Figure S15. ^{13}C-NMR of Fmoc-Sez-OH

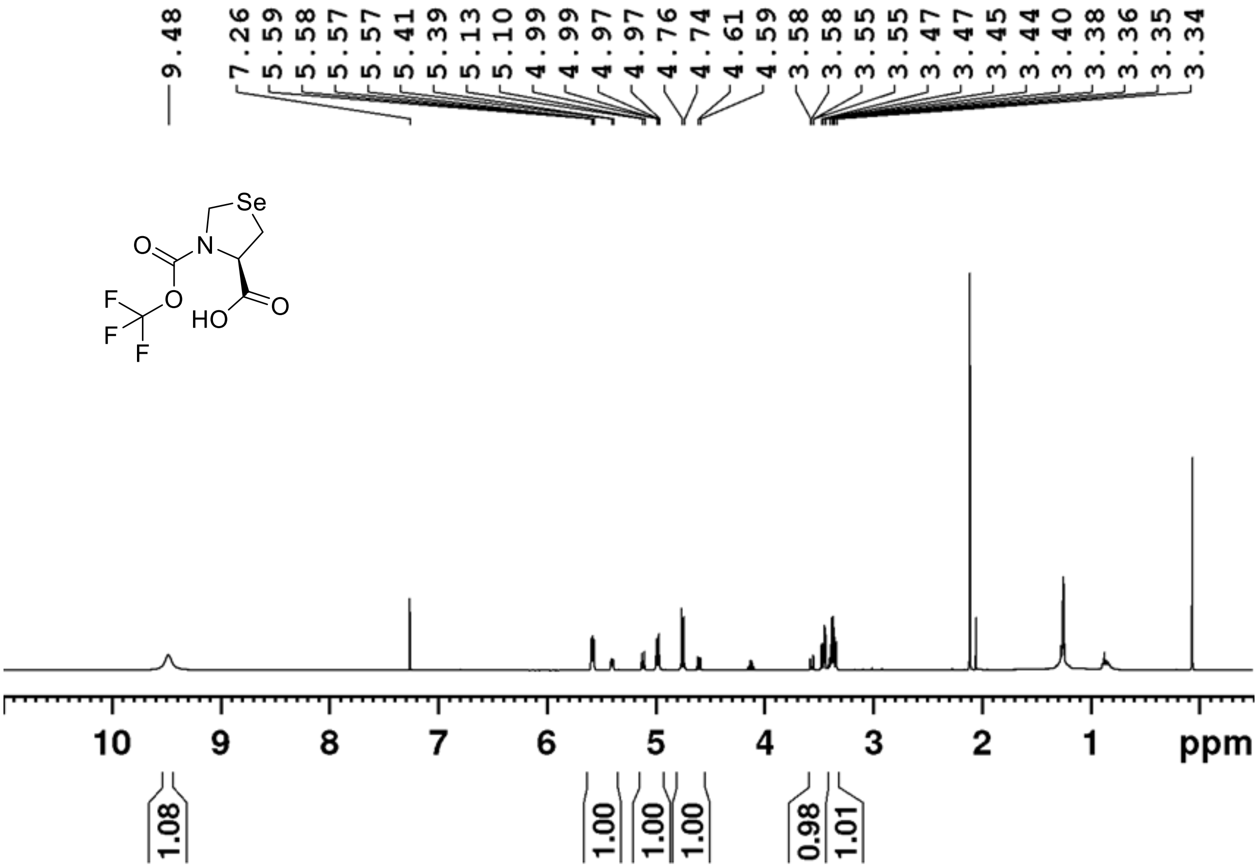

Figure S16. ^{1}H-NMR of Tfa-Sez-OH

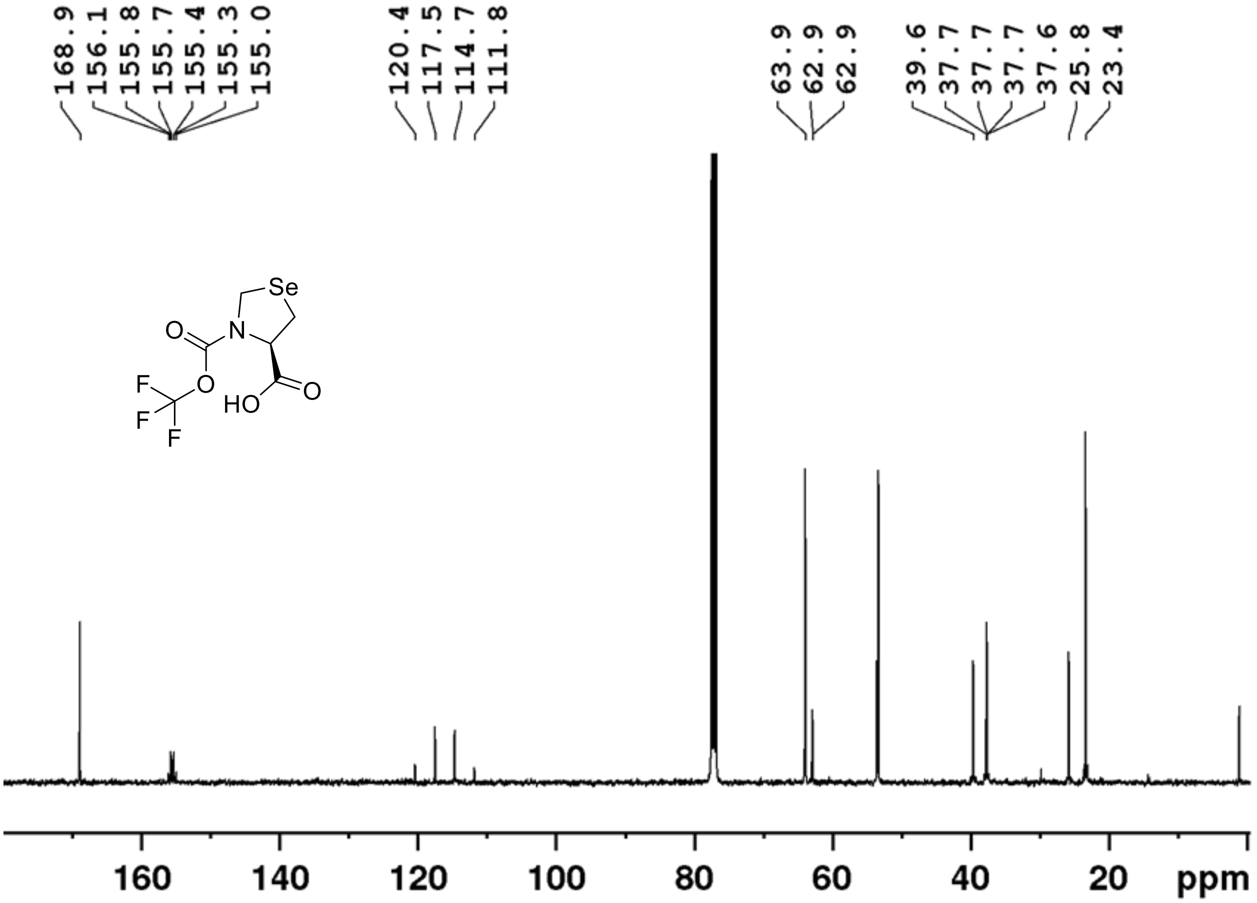

Figure S17. ^{13}C-NMR of Tfa-Sez-OH

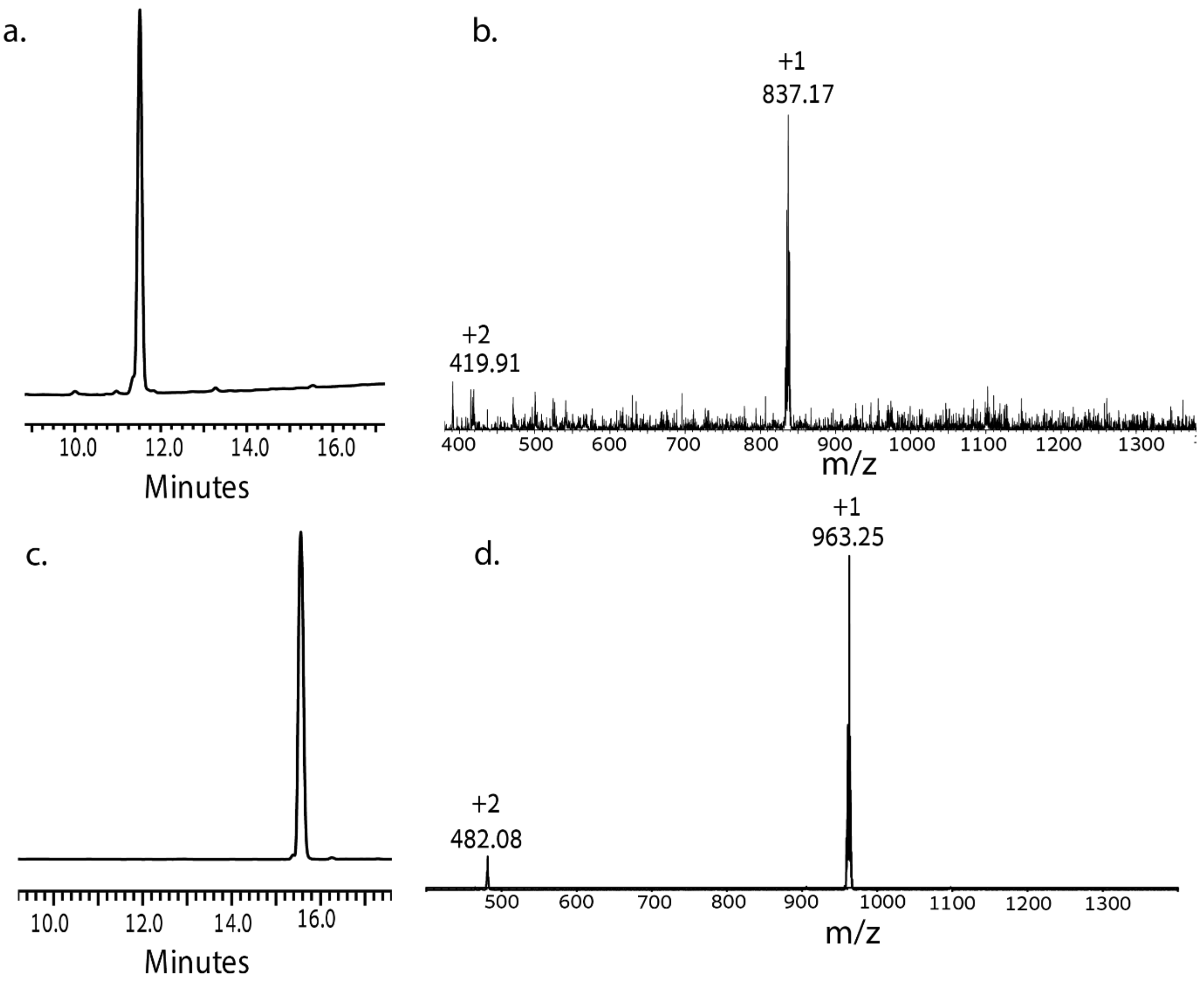

Figure S18. Characterization of the model peptide segment. a. Analytical HPLC analysis (220 nm) of Tfa-Sez-LYRAG-NH_2; b. The corresponding ESI-MS, with its deconvoluted mass (inset) (obs. 837.17 ± 0.46 Da, calc. 836.27 Da) a. Analytical HPLC analysis (220 nm) of Fmoc-Sez-LYRAG-NH_2; b. The corresponding ESI-MS, with its deconvoluted mass (inset) (obs. 963.25 ± 0.77 Da, calc. 962.36 Da).

6. References

[1] P. S. Reddy, S. Dery, N. Metanis, *Angew. Chem. Int. Ed.* **2016**, *55*, 992–995.

[2] M. D. Gieselman, L. Xie, W. A. Van Der Donk, *Org. Lett.* **2001**, *3*, 1331–1334.

[3] A. L. Schroll, R. J. Hondal, S. Flemer, *J. Pept. Sci.* **2012**, *18*, 155–162.

[4] J. S. Zheng, S. Tang, Y. K. Qi, Z. P. Wang, L. Liu, *Nat. Protoc.* **2013**, *12*, 2483–2495.

[5] P. E. Flood, D. T. , Hintzen, J. C.J., Bird, M. J., Cistrone, P. A., Chen, J. S. & Dawson, *Angew. Chem. Int. Ed.* **2018**, *57*, 11634–11639.

[6] S. K. Mahto, C. J. Howard, J. C. Shimko, J. J. Ottesen, *ChemBioChem* **2011**, *12*, 2488–2494.

[7] N. Thieriet, P. Gomez-Martinez, F. Guibé, *Tetrahedron Lett.* **1999**, *40*, 2505–2508.

[8] J. B. Blanco-Canosa, P. E. Dawson, *Angew. Chem. Int. Ed.* **2008**, *47*, 6851–6855.

[9] N. Dery, S., Post, S., Dery, L., Mousa, R., Notis Dardashti, R., and Metanis, *Chem. Sci.* **2015**, *6*, 6207–6212.

[10] L. R. Malins, K. M. Cergol, R. J. Payne, *Chembiochem* **2013**, *14*, 559–563.

[11] A. Kar, A., Mannuthodikayil, J., Singh, S., Biswas, K. Dubey, P., Das, A. & Mandal, *Angew. Chem. Int. Ed.* **2020**, *59*, 14796–14801.

[12] J. Lundström, A. Holmgren, *Biochemistry* **1993**, *32*, 6649–6655.

[13] F. Aslund, K. D. Berndt, A. Holmgren, *J. Biol. Chem.* **1997**, *272*, 30780–30786.

[14] N. Metanis, E. Keinan, P. E. Dawson, *J. Am. Chem. Soc.* **2006**, *128*, 16684–16691.

[15] A. Holmgren, *J. Biol. Chem.* **1979**, *254*, 9627–9632.

[16] J. Y. Chang, P. Schindler, B. Chatrenet, *J. Biol. Chem.* **1995**, *270*, 11992–7.

[17] J. s. Weissman, P. s. Kim, *Science* **1991**, *253*, 1386–1393.
